# A randomized, double blind, placebo controlled, multicenter clinical trial to assess the efficacy and safety of *Emblica officinalis* extract in patients with dyslipidemia

**DOI:** 10.1186/s12906-019-2430-y

**Published:** 2019-01-22

**Authors:** Haridas Upadya, S. Prabhu, Aravinda Prasad, Deepa Subramanian, Swati Gupta, Ajay Goel

**Affiliations:** 1 Aadhitya Adhikari Hospital, Contour Road, Gokulam, Mysore, 570002 India; 2 LifeCare Hospital, No.99, OM Complex, 20th Main, Gangothri Circle, BTM 1st Stage, Bangalore, 560029 India; 3 Sri Venkateshwara Hospital, No.86, Hosur Main Road, Madiwala, Bangalore, 560068 India; 4 Syncretic Clinical Research Services, No. 4, 5th cross, 11th Main Road, Vasanthnagar, Bangalore, 560052 India; 5 Amrita School of Pharmacy, Amrita Institute of Medical Sciences and Research Centre, Amrita Vishwa Vidyapeetham, Kochi, 682041 India; 6 Center for Gastrointestinal Research, Center for Translational Genomics and Oncology, Baylor Scott & White Research Institute and Sammons Cancer Center, Baylor University Medical Center, Dallas, Texas, 3410 Worth Street, Suite 610, Dallas, TX 75246 USA

**Keywords:** *Emblica officinalis*, Cholesterol, AIP, TG, CoQ10, Dyslipidemia

## Abstract

**Background:**

Dyslipidemia is one of the most frequently implicated risk factors for development of atherosclerosis. This study evaluated the efficacy of amla (*Emblica officinalis*) extract (composed of polyphenols, triterpenoids, oils etc. as found in the fresh wild amla fruit) in patients with dyslipidemia.

**Methods:**

A total of 98 dyslipidemic patients were enrolled and divided into amla and placebo groups. Amla extract (500 mg) or a matching placebo capsule was administered twice daily for 12 weeks to the respective group of patients. The patients were followed up for 12 weeks and efficacy of study medication was assessed by analyzing lipid profile. Other parameters evaluated were apolipoprotein B (Apo B), apolipoprotein A1 (Apo A1), Coenzyme Q10 (CoQ10), high-sensitive C-reactive protein (hsCRP), fasting blood sugar (FBS), homocysteine and thyroid stimulating hormone (TSH).

**Results:**

In 12 weeks, the major lipids such as total cholesterol (TC) (*p* = 0.0003), triglyceride (TG) (*p* = 0.0003), low density lipoprotein cholesterol (LDL-C) (*p* = 0.0064) and very low density lipoprotein cholesterol (VLDL-C) (*p* = 0.0001) were significantly lower in amla group as compared to placebo group. Additionally, a 39% reduction in atherogenic index of the plasma (AIP) (*p* = 0.0177) was also noted in amla group. The ratio of Apo B to Apo A1 was reduced more (*p* = 0.0866) in the amla group as compared to the placebo. There was no significant change in CoQ10 level of amla (*p* = 0.2942) or placebo groups (*p* = 0.6744). Although there was a general trend of FBS reduction, the numbers of participants who may be classified as pre-diabetes and diabetes groups (FBS > 100 mg/dl) in the amla group were only 8. These results show that the amla extract used in the study is potentially a hypoglycaemic as well. However, this needs reconfirmation in a larger study.

**Conclusions:**

The Amla extract has shown significant potential in reducing TC and TG levels as well as lipid ratios, AIP and apoB/apo A-I in dyslipidemic persons and thus has scope to treat general as well as diabetic dyslipidemia. A single agent to reduce cholesterol as well as TG is rare. Cholesterol reduction is achieved without concomitant reduction of Co Q10, in contrast to what is observed with statins.

**Trial registration:**

Registered with Clinical Trials Registry- India at www.ctri.nic.in (Registration number: CTRI/2015/04/005682) on 8 April 2015 (retrospectively registered).

**Electronic supplementary material:**

The online version of this article (10.1186/s12906-019-2430-y) contains supplementary material, which is available to authorized users.

## Background

Dyslipidemia is one of the major risk factors for development of atherosclerosis and other heart disorders [1]. This indicates a high level of lipids in the blood, of which includes triglycerides (TGs) and total cholesterol (TC). All over the world including Indian population, both genetic disorders and diets high in saturated fats and cholesterol, contribute to elevated lipid levels [2].A lot of efforts are being made worldwide to diagnose cardiovascular disease. The conventional lipid parameters include triglycerides (TGs), total cholesterol (TC), high density lipoproteins (HDL), low density lipoproteins (LDL) and very low density lipoproteins (VLDL). It is thus apparent that for effective management of dyslipidemia, several of the individual parameters need to be controlled simultaneously. This is amply demonstrated by the observation that people whose cholesterol has been brought to normal levels are not free from CVD risk. Several lipoprotein ratios have also been defined to optimize the predictive capacity of the lipid profile. Atherogenic index of plasma (AIP) is one such measure and could prove to be a better alternative to the simple lipid profile [3]. AIP is the logarithmically transformed ratio of molar concentrations of TGs to high density lipoproteins (HDL) ((log (TG/HDL [mmol])). AIP is increased in people at higher risk for coronary heart disease and has recently been identified as a marker of plasma atherogenicity. This simple ratio reflects the balance between atherogenic versus antiatherogenic lipids and is inversely correlated with LDL-C particle size. AIP is also associated with prediction of type 2 diabetes mellitus (T2DM). A recent meta-analysis of 15 studies suggest that conventional plasma lipid parameters have ability to predict the risk of T2DM, but AIP is the most important among all and may be more closely associated with the risk of T2DM. Almost all the studies reported in the meta-analysis reported positive associations between AIP and T2DM [4].

The apolipoproteins play very important role in lipoprotein metabolism [5]. The largest component of the HDL is apolipoprotein A1 (Apo A1). It’s major role is acting as a mediator in transfer of cholesterol from cells to HDL particles. It also works as co-factor for lecithin cholesterol acyl transferase enzyme. Both of these two processes are very important for the reverse transport of cholesterol to the liver [6]. Apolipoprotein B (Apo B) is another important apolipoprotein which is present in chylomicrons, VLDL, IDL and LDL [7]. It is considered as the major functional protein which transports cholesterol to peripheral cells [8]. There is strong association between the risk of CVD and increased Apo B/Apo A1 ratios [9]. There are several prospective studies such as the AMORIS [10], INTERHEART [11], EPIC-Norfolk [12], ULSAM [13], and the MONICA/KORA [14] which confirms the importance of Apo B/Apo A1 ratios in predicting CVD. In one study with non-diabetic subjects, Apo B/Apo A-I ratio has been shown to be an independent predictor of insulin resistance [15]. The utility of this ratio is further supported by a meta-analysis as a future risk marker of CVD [16]. Conventional lipid indices alone may result in erroneous conclusions in the assessment of CVD risk [17]. A proportion of the subjects with accepted range of lipid parameters [18] and some patients who achieve significant decrease in LDL cholesterol levels with lipid lowering treatment [19] still develop atherosclerotic disease. Lipoprotein cholesterol concentration may not always correspond to lipoprotein concentration [20]. This discrepancy can be corrected by the apoB/apoA-1 ratio and discriminate between patients with CVD and those without even when they have normal lipid levels [21].

Various medicinal plants used in the traditional systems of medicine have shown potential in the management of dyslipidemia and CVD without major adverse events. *Emblica officinalis* (Amla or Indian gooseberry) is one such medicinal plant which has been shown to be effective in the management of dyslipidemia in experimental animals and in pilot clinical studies without major side effects [22, 23]. In vitro and animal studies have shown that the flavonoids from *Emblica officinalis* effectively reduce lipid levels in blood serum and tissues [24]. In a study in rabbits, amla extract was found effective in the management of dyslipidemia by reducing LDL and increasing HDL levels. The authors also reported that intima media thickening got reversed on administration of amla extract [25]. Few more reports are published on hypolipidemic effects of amla on cholesterol induced atherosclerosis in rabbits [26, 27]. In a pilot clinical study by Antony et al., Emblica officinalis extract at a dose of 500 mg and 1000 mg per day, showed a significant reduction in TC and TG and a significant increase in HDL. The inflammatory marker C-reactive protein (CRP) was also significantly reduced [28]. In this study the extract was aqueous extract of fresh amla fruits without seeds and standardized to 35% galloelagi tannins. In another study by same group, 50% methanol extract of amla at 500 mg twice daily reduced the TC, TG and LDL significantly and increased the HDL as compared to control group [29]. Another clinical study comparing the efficacy and safety of amla capsule (500 mg/day) with simvastatin (20 mg/day) has shown that amla produced significant reduction of TC, LDL, TG and VLDL, and a significant increase in HDL [30].

In all the above reported trials with amla extract, the main focus of researchers was on measurement of lipid profile only. Moreover, the amla extract used in above studies was made without using the amla seeds. The amla extract used in the present study is full spectrum amla fruit extract including the extract from amla seeds and amla fruit pulp. Furthermore, apart from conventional lipid parameters, we have also measured the AIP, apoB/apoA-1, CoQ10 level, TSH etc. which may provide a more conclusive prediction on heart health than lipid profile alone.

The primary objective of the present study was to assess the effect of amla extract on TG levels. Additionally, we also studied the AIP and apoB/apoA-1 ratios as reliable risk factors for CVD in patients suffering from dyslipidemia. Another objective of this study was to assess the safety of amla extract with emphasis on the side effects observed in conventional lipid lowering therapies.

This report pertains to the results of a randomized, multicentre study using an extract of amla (Indian gooseberry, *Emblica officinalis*) fruits to effectively reduce high TC and TG levels in persons. The extract also reduces apoB/apoA-I ratios potentially indicating its utility in treating diabetic dyslipidemia, in particular. Amla belongs to a group of plants classified as *Rasayana* plants under the Ayurvedic system of traditional medicine. Rasayana plants are known for their all-round health benefits and outstanding safety profiles.

## Methods

### Study medication and placebo

A 500 mg capsule of amla extract (Arjuna Natural Ltd., Aluva, Kerala, India) was used for the intervention group. Amla extract was prepared by extracting fresh whole fruits of amla with ethyl acetate and standardized to contain not less than 35% polyphenols, 8% triterpenoids and 10% oil. The extract also contains Omega 3 fatty acids. This is the first report on amla extract containing omega 3 fatty acids. The details on characterization of the amla extract used in the study are provided in the Additional file 1. Each 500 mg of amla extract capsule contains about 175 mg polyphenols, 40 mg triterpenoids and 50 mg oil. Similarly, 500 mg placebo capsules (roasted rice powder) were used for control group. Vegetarian capsule shells of size ‘0’ were filled with 500 mg amla extract powder (intervention group) or roasted rice powder (placebo). Semi-automatic capsule filling machine was used to fill the required number of capsules.

### Impact of roasted rice (as placebo) in patients with dyslipidemia

In the present study, roasted rice powder used as a placebo was given at a dosage of 500 mg capsule twice daily for 12 weeks. Rice is known as the grain of life and is synonymous with food for every Indian. It is the staple food for two thirds of the Indian population [31]. The Indians derive 80% of their energy needs from rice, which contains about 80–90% carbohydrates, 7–8% protein, 2–3% fat, and 1–2% fiber [32]. The major component of rice responsible for its medicinal value is the rice bran [33, 34]. It is reported that, higher daily intakes of rice bran for longer duration is needed to induce favorable changes in serum lipid parameters [34]. The roasted rice powder used in the present study was prepared by taking white rice devoid of any husk, bran or germ. The dose was only 500 mg twice daily which is very less compared to serving of rice as a regular meal. Thus, there was very negligible chance of any impact of roasted rice powder on lipid profile of patients with dyslipidemia.

### Chemicals

Kits for TC, TG, LDL-C, HDL-C, VLDL-C, Apo A1, Apo B, TSH, plasma glucose, SGOT (serum glutamic oxaloacetic transaminase), SGPT (serum glutamic pyruvic transaminase), bilirubin, creatinine and blood urea nitrogen (BUN) were purchased from Siemens Limited, Mumbai, India. Human Coenzyme Q10 (CoQ10) kit was purchased from Elabscience, USA. All other chemicals were of analytical grade and purchased locally.

### Study design

**Fig. 1:**
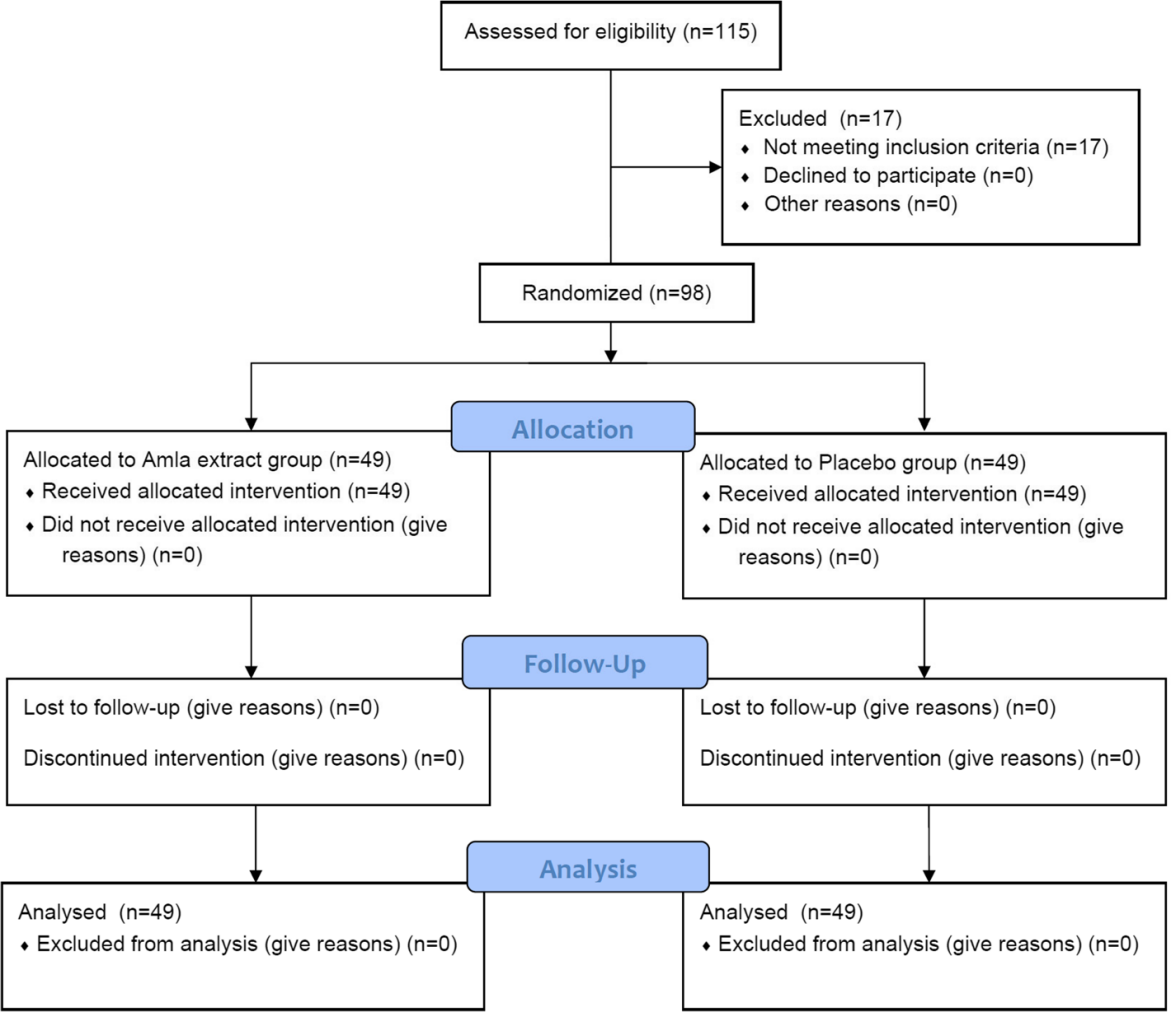
The CONSORT Diagram

**Fig. 2:**
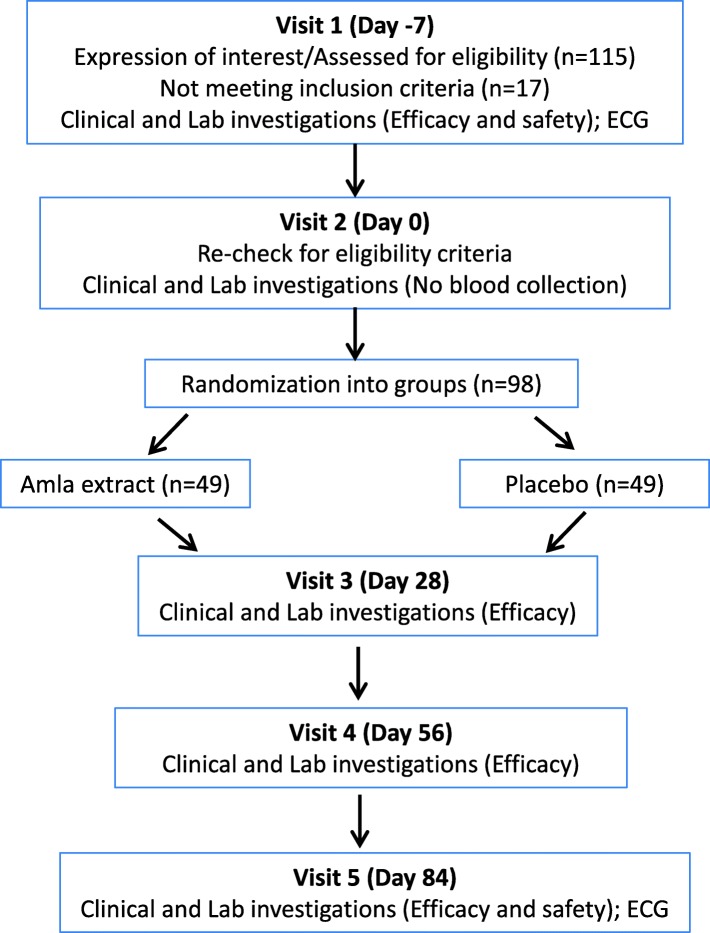
Systemic illustration of study design

The study was a multicenter (a total of four study centers across South India; Additional file 2), randomized, double blind, parallel, placebo controlled, clinical trial to evaluate the efficacy and safety of amla extract 500 mg, compared to a placebo in patients with dyslipidemia (study protocol included as Additional file 3). The study followed CONSORT guidelines and criteria as illustrated in Fig. 1 and the Additional file 4. This was a parallel treatment study of 12 weeks duration. There were a total of 5 visits (Fig. 2): Visit 1 -Screening (Day − 7), Visit 2, Randomization (Day 0), Visit 3, Follow up (Day 28 ± 5 days), Visit 4, Follow up (Day 56 ± 5 days) and Visit 5, End of study visit (Day 84 ± 5 days). Laboratory investigation of all efficacy parameters were conducted at screening, on all follow up visits and end of study visit while laboratory assessments for safety parameters were performed on Visit 1 (screening) and Visit 5 (end of the study). The study protocol was approved by respective institutional ethics committees, i.e. Mysore Clinical Research Ethics Committee on 22/2/2015 (study site: Aadhitya Adhikari Hospital, Contour Road, Gokulam, Mysore-570,002), Life Care Hospital Institutional Ethics Commitee on 15/3/2015 (study site: LifeCare Hospital, 99, OM Complex, 20th Main, Gangothri Circle, BTM 1st Stage, Bangalore-560,029), Sri Venkateshwara Hospital Ethics Committee on 15/3/2015 (Study site: Sri Venkateshwara Hospital, No.86, Hosur Main Road, Madiwala, Bangalore- 560,068) and Prashanth Hospital Ethics Committee on 17/3/2015 (study site: Prashanth Hospital, Bommanahalli Circle, Hosur Main Road, Bangalore-560,068) and and registered with Clinical Trials Registry- India at www.ctri.nic.in (Registration number: CTRI/2015/04/005682) on 8 April 2015 (retrospectively registered).

To maintain blindness of study treatments, placebo capsules resembling in every manner to amla extract capsule were used. Sealed envelopes/scratch cards were provided to the investigators by the sponsor (one each for each randomization number) indicating the randomization number and the test product contained therein. The sealed envelopes/scratch cards were kept by the investigator in a safe but accessible place should the need for breaking the blinding medication code arises in an emergency.

### Sample size

Sample size was determined for t- test. For getting a power of 90%, 39 patients would be needed in each group. Assuming the dropout, missing data etc., of 20%, a total of 98 patients were enrolled comprising of 49 in each group.

### Study population

A total of 98 patients, male and female, at multiple centers were simultaneously enrolled in this clinical trial. The enrolled patients were assigned to either of the two study groups according to the centralized computer generated randomization in a 1:1 ratio using Graph Pad Prism software. The study was conducted during March 2015 to November 2015.

### Inclusion and exclusion criteria

Any study related activity was undertaken only after subjects voluntarily signed the consent form. Patients (male/female) between 30 and 65 years of age having TG > 200 mg/dL, LDL-C > 130 mg/dL, TC > 200 mg/dL and HDL-C < 40 mg/dL for men and < 50 mg/dL for women were included in the study. Patients were not taking any medication (including herbal product) for management of dyslipidemia since the last 4 weeks.

Patients with more than 2 of the following risk factors: Cigarette smoking, Hypertension (BP > 140/90 mmHg or on antihypertensive medication), Family history of premature CHD (CHD in male first degree relative < 55 years; CHD in female first degree relative < 65 years), Age (men > 45 years; women > 55 years) were excluded from the study. Patients with uncontrolled cardiovascular disease or advanced atherosclerosis (e.g. history of stroke, myocardial infarction, life-threatening arrhythmia, or coronary revascularization within the preceding 6 months; unstable angina; congestive heart failure; known or suspected clinically significant valvular heart disease or uncontrolled hypertension (> 160/100 mm of Hg or use of antihypertensive medications, dose of which is not stable in the last 1 month) were also excluded. Patients with TG levels > 500 mg/dL or FPG more than 150 mg/dL, using insulin, glitazones, or other hypoglycemics, at a dose of which was not stable in last 1 month were also excluded.

Pregnancy, lactation and female patients not using acceptable contraceptive measures (double barrier methods, oral or injectable hormonal contraceptives or surgical sterilization), patients with hepatic impairment (SGOT or SGPT levels > 3 Upper Limit of Normal (ULN)) or renal impairment (serum creatinine ≥2.0 mg/dl), patients with any other severe systemic illness, those that in the opinion of the investigator would be noncompliant with the visit schedule or study procedures and patients with known history of hypersensitivity to Amla or any product containing Amla extract were also excluded. Patients with continuing history of alcohol and/or drug abuse, patients with any other serious concurrent illness or malignancy, or participating in another clinical trial in the past 3 months were also excluded.

### Study procedure

The study was a double blind investigation, randomizing eligible patients using a centralized computer generated randomization plan to receive either 500 mg amla extract capsule or the corresponding placebo twice daily (after breakfast in the morning and after dinner in the evening) for 12 weeks. All the patients enrolled in the study were also asked to initiate lifestyle changes (healthy diet with exercise at least 4 days a week) along with the study medication.

All patients were followed up on an outpatient basis for a period of 84 days ±5 days (12 weeks) with scheduled visits on day 28, day 56 and day 84 post-randomization. Efficacy assessments were carried out by laboratory investigations on each of the scheduled visits (except the randomization visit). Clinical adverse events monitoring was done on each visit and complete laboratory check-ups (both haematological and biochemical) and ECG were repeated at the end of the study (12 weeks), so as to assess the safety of the study medication. Laboratory values obtained during the screening/randomization visit were considered as baseline values for efficacy and safety analysis.

### Assessment of efficacy

The venous blood samples were collected from the participants during each visit after overnight (12 h) fasting to measure the efficacy and safety parameters. Efficacy of the study medication was assessed by the primary serum lipid parameters: TG, TC, LDL-C, VLDL-C and HDL-C. The AIP was calculated by taking logarithm to the base 10 (Common Logarithm) of the ratio of the molar concentration of TG to HDL-C [35]. Other important parameters measured were Apo A1 & Apo B and Coenzyme Q10 (CoQ10). The ratio of Apo B to Apo A1 was calculated. Other related parameters like fasting plasma glucose (FPG), homocysteine levels, and TSH were also analyzed. The tests were conducted as per procedure available with the respective kits. The treatment compliance was calculated on the basis of number of capsules unit left in the bottle at each follow up visits.

### Statistical analysis

Statistical analysis was conducted using SPSS version 24.0 for Windows. Continuous variables were expressed as means and standard deviations and discrete variables were expressed as proportions. Chi square test was used for comparing the proportions. Normally distributed data were analysed using independent t-test to determine the statistical significance between groups and paired t-test was used to analyse within the group. For non-normally distributed data were analysed using Mann-Whitney to find the significance between the groups and and within the groups for such data Wilcoxon signed rank test was used. Additionally ANOVA with repeated measure is done after doing Mauchly’s test for sphericity. If Mauchly’s test statistic is non-significant (*p* > 0.05) it is reasonable to conclude that the variances of differences are not significantly different (i.e. they are roughly equal) and sphericity is assumed, otherwise sphericity is not met and Greenhouse-Giesser values are used to calculate the F-statistics and corresponding eta-square is calculated. A value of eta-square 0.01–0.06 is considered as small effect, 0.06–0.14, medium effect and above 0.14 large effect. The mean scores for clinical parameters were statistically significantly different if *p* value for F statistics were less than 0.05. The mean difference of each parameter between different time point were calculated and if *p* < 0.05 the change is significant between different time points. Single tailed, Wilcoxon signed rank test, Wilcoxon Rank Sum Test (Mann–Whitney) and ANCOVA were used for statistical analysis of clinical parameters to compare the groups and change within the group between the base line data and final data. A p value of less than 0.05 was considered as significant. The power of the study is 80% as the mean reduction of the primary end point (TG) and SD of TG did not vary much from the initially assumed values taken at the time of study design.

## Results

### Demographic details and baseline data

A total of 98 patients (45 males and 53 females) were enrolled and all the patients completed the study at multiple centers in India. The patient demographic characteristics are shown in Table 1, and indicate that the study population was homogenous, with no statistically significant differences between the groups with respect to demographic variables. There was no dropout reported in the study. The vital parameters i.e. blood pressure, heart-rate, axillary temperature, respiratory rate, etc. were in normal range before start of the study. There was no significant difference in vital parameters at baseline and at the end of study (visit 5) in amla extract as well as the placebo group.

**Table 1:** Subject’s baseline data at the start of the study

Parameters	Patients treated with Amla Extract (Mean ± S.D)	Patients treated with Placebo (Mean ± S.D)	*p*-value
Age (years)	40.7 ± 10.13	42.2 ± 9.20	0.44
Height (cm)	162.7 ± 9.20	163.1 ± 9.54	0.82
Weight (kg)	70.4 ± 10.57	69.8 ± 9.04	0.75
Number of participants	49	49	
Number of male subjects	22	23	0.84
Number of female subjects	27	26	
Body Mass Index (BMI; Kg/m^2^)	26.9 ± 3.77	26.1 ± 3.39	0.27
Systolic Blood Pressure (mmHg)	118.1 ± 5.77	119.7 ± 5.19	0.15
Diastolic Blood Pressure (mmHg)	77.9 ± 6.87	77.4 ± 7.06	0.72
Heart Rate (Beats per minute)	77.5 ± 6.11	77.1 ± 6.52	0.75
Axillary Temperature (degrees Celcius)	36.96 ± 0.243	36.94 ± 0.308	0.72
Total cholesterol (mg/dL)	231.7 ± 27.03	225.7 ± 29.03	0.27
Triglycerides (mg/dL)	261.1 ± 74.13	247.6 ± 57.70	0.84
High Density Lipoproteins (mg/dL)	43.7 ± 7.45	43.4 ± 7.34	0.09
Low Density Lipoproteins (mg/dL)	140.0 ± 19.66	132.2 ± 20.82	0.06
Very Low Density Lipoproteins (mg/dL)	51.7 ± 15.9	49.6 ± 11.68	0.70
Atherogenic index of Plasma	0.43 ± 0.14	0.41 ± 0.12	0.82
Fasting Plasma Glucose (mg/dL)	90.6 ± 26.83	91.9 ± 22.12	0.44
Ratio of Apo B to Apo A1	1.0 ± 0.34	0.87 ± 0.22	0.35
CoQ10 (ng/ml)	471.5 ± 183.5	487.0 ± 167.5	0.27
HMGCoA (ng/ml)	62.4 ± 11.06	53.4 ± 7.07	0.48
Glycolated Hb	6.25 + 1.05	6.22 + 1.11	0.47
Homocystiene (μmol/L)	23.58 ± 14.57	21.4 ± 11.10	0.36
TSH (μIU/ml)	2.43 ± 1.50	2.53 ± 2.58	0.33
CRP	3.71 + 5.82	4.58 + 5.96	0.10
Blood Urea (mg/dl)	9.7 ± 3.42	10.2 ± 5.39	0.93
Hb	14.03 ± 2.34	13.89 ± 2.04	0.48
Serum Creatinine-(mg/dl)	0.8 ± 0.15	0.9 ± 0.60	0.64
Serum Bilirubin-(mg/dl)	0.6 ± 0.32	0.6 ± 0.28	0.28
SGOT (U/L)	27.2 ± 10.23	27.4 ± 9.35	0.98
SGPT (U/L)	38.6 ± 20.86	37.2 ± 18.56	0.88

### Triglyceride, HDL-C and Atherogenic index of plasma

**Fig. 3:**
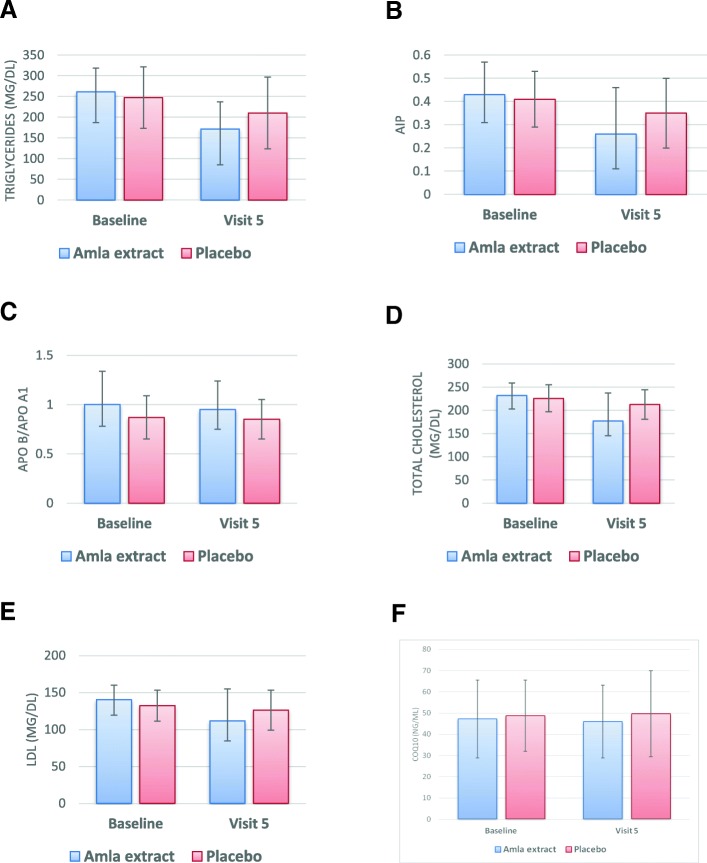
(**a**) Triglyceride level in amla extract and placebo group (*p* = 0.0003 between the groups); (**b**) Atherogenic index of plasma in amla extract and placebo group (*p* = 0.0177 between the groups); (**c**) Ratio of Apo B to Apo A1 at baseline and visit 5 in amla extract and placebo group (*p* = 0.0866 between the groups); (**d**) TC level at baseline and visit 5 in amla extract and placebo group (*p* = 0.0003 between the groups); (**e**) LDL-C level at baseline and visit 5 in amla extract and placebo group (*p* = 0.0064 between the groups); (**f**) CoQ10 level at baseline and visit 5 in amla extract and placebo group (*p* = 0.4581 between the groups)

There was a highly significant reduction in TG from 261.11 ± 74.13 mg/dl (baseline) to 171.94 ± 86.51 mg/dl (visit 5) in the amla extract group (*p* < 0.0001 within the group), whereas the TG reduction in the placebo group was from 247.62 ± 57.70 mg/dl to 210.47 ± 65.27 mg/dl (Fig. 3a). This reduction in TG was also highly significant (*p* = 0.0003) between the groups. AIP was reduced from 0.43 ± 0.14 to 0.26 ± 0.20 in amla extract group (*p* < 0.0001 within the group) whereas reduction was from 0.41 ± 0.12 to 0.35 ± 0.15 in the placebo group (Fig. 3b). The change in AIP was also highly significant between the two groups (*p* = 0.0177). The change in HDL from baseline to visit 5 was not significant between the groups. The complete data for all the visits is available as Additional file 5.

### Ratio of Apo B to Apo A1

The data for the ratio of Apo B to Apo A1 is presented in Fig. 3c. The ratio of Apo B to Apo A1 significantly reduced from 1.00 ± 0.34 to 0.94 ± 0.29 in amla extract group (*p* = 0.0419 within the group) whereas in placebo group the ratio was decreased (non-significant) from 0.87 ± 0.22 to 0.85 ± 0.20 (*p* = 0.6434 within the group). There was no significant difference between the groups (*p* = 0.0866) in ratio of Apo B to Apo A1.

### TC, LDL-C and VLDL-C level

In the amla extract group, the TC was significantly reduced from 231.67 ± 27.03 mg/dl (baseline) to 177.00 ± 60.04 mg/dl (visit 5) after 12 weeks of treatment (*p* < 0.0001 within the group). In the placebo group, TC was reduced from 225.74 ± 29.03 mg/dl (baseline) to 212.55 ± 31.78 mg/dl (*p* = 0.0098 within the group). The reduction in TC was highly significant (*p* = 0.0003) between amla extract and placebo group (Fig. 3d). LDL-C was significantly reduced from 139.96 ± 19.66 mg/dl to 111.53 ± 43.22 mg/dl in amla extract group (*p* < 0.0001 within the group) whereas in placebo group the baseline LDL-C was 132.15 ± 20.82 mg/dl and reduced to 126.06 ± 27.02 mg/dl at the end of study (*p* = 0.1008 within the group). This reduction in LDL-C was also highly significant (*p* = 0.0064) between the two groups (Fig. 3e). VLDL-C was also reduced in both the groups after 12 weeks treatment. In amla extract group the VLDL-C was significantly reduced from 50.56 ± 15.9 mg/dl to 33.04 ± 17.32 mg/dl (*p* < 0.0001 within the group) whereas in placebo group, the change was from 50.72 ± 11.68 mg/dl to 42.79 ± 12.89 mg/dl (*p* = 0.0003 within the group). The reduction in VLDL-C was also highly significant (*p* = 0.0001) between the two groups.

### CoQ10 level

As presented in Fig. 3f, there was a very slight change (not significant; *p* = 0.2942 within the group) in CoQ10 level in the amla extract group from 47.15 ± 18.27 ng/ml (baseline) to 45.98 ± 17.15 ng/ml (visit 5), whereas change in the placebo group was from 48.69 ± 16.75 ng/ml to 49.60 ± 20.32 ng/ml (*p* = 0.6744 within the group). The change in CoQ10 level was not statistically significant between the groups (*p* = 0.4581) or between baseline and at the end of the study.

### Other efficacy parameters

The homocysteine level was reduced in the amla extract group from 23.58 ± 14.57 μmol/L (baseline) to 19.17 ± 8.93 μmol/L (visit 5), whereas the reduction in the placebo group was from 21.4 ± 11.10 μmol/L to 17.18 ± 5.8 μmol/L. The change in homocysteine level was not statistically significant between the groups (*p* > 0.05). There was an increase in the TSH level of amla extract as well as placebo groups when baseline values were compared with visit 5 values and the change was more in placebo group. In this case also the increase in TSH level was not statistically significant between the groups. The change in FPG level was also not significant between amla extract and placebo groups. The *p* values within the group and between groups are mentioned in Table 2 for all the parameters.

**Table 2:** Table with p values within the group (amla extract/placebo) and between groups

Triglycerides (mg/dl)						
	Placebo		Amla extract		Between groups (MW)	
	Initial	Final	Initial	Final	Initial	Final
Mean TG levels (mg/dl)	247.617	210.469	261.106	171.939		
p (within group) (W)	*p* = 0.0007		*p* < 0.0001		*p* = 0.8367	*p* = 0.0003
Decrease in TG (n)	35		44			
Increase in TG (n)	14		4			
No change (n)	0		1			
p (chi-square)	*p* = 0.021					
Total Cholesterol (mg/dl)						
	Placebo		Amla extract		Between groups (MW)	
	Initial	Final	Initial	Final	Initial	Final
Mean TC levels (mg/dl)	225.735	212.551	231.673	177		
p (within group) (W)	*p* = 0.0098		*p* < 0.0001		*p* = 0.2707	*p* = 0.0003
Decrease in TC (n)	31		40			
Increase in TC (n)	16		9			
No change	2		0			
p (chi-square)	*P* = 0.129					
Low Density Lipoproteins (mg/dl)						
	Placebo		Amla extract		Between groups (MW)	
	Initial	Final	Initial	Final	Initial	Final
Mean LDL levels (mg/dl)	132.155	126.059	139.963	111.531		
p (within group) (W)	*p* = 0.1008		*p* < 0.0001		*p* = 0.0404	*p* = 0.0064
Decrease in LDL (n)	28		36			
Increase in LDL (n)	20		13			
No change (n)	1		0			
p (chi-square)	*P* = 0.174					
High Density Lipoproteins (mg/dl)						
	Placebo		Amla extract		Between groups (MW)	
	Initial	Final	Initial	Final	Initial	Final
Mean HDL levels (mg/dl)	43.418	41.659	43.657	39.339		
p (within group) (W)	*p* = 0.2849		*p* = 0.0002		*p* = 0.5527	*p* = 0.0149
Decrease in HDL (n)	28		36			
Increase in HDL (n)	21		12			
No change (n)	0		1			
p (chi-square)	*p* = 0.101					
Atherogenic Index of Plasma						
	Placebo		Amla extract		Between groups	
	Initial	Final	Initial	Final	Initial (MW)	Final (T-test)
Mean AIP level	0.410	0.347	0.426	0.261		
p (within group) (W)	*p* = 0.0098		*p* < 0.0001		*p* = 0.8119	*p* = 0.0177
Decrease in AIP (n)	31		40			
Increase in AIP (n)	18		8			
No change (n)	0		1			
p (chi-Square)	*p* = 0.045					
CoQ10 (ng/ml)						
	Placebo		Amla extract		Between groups	
	Initial	Final	Initial	Final	Initial (MW)	Final (T-test)
Mean CoQ10 levels (ng/ml)	48.691	49.602	47.146	45.975		
p (within group)	*p* = 0.6744 (t-test)		*p* = 0.2942 (W)		*p* = 0.7788	*p* = 0.4581
Decrease in CoQ10 (n)	14		18			
Increase in CoQ10 (n)	16		12			
No change (n)	0		0			
p (chi-Square)	*p* = 0.438					
Ratio of Apo B/Apo A1						
	Placebo		Amla extract		Between groups (MW)	
	Initial	Final	Initial	Final	Initial	Final
Mean value	0.869	0.853	1.004	0.948		
p (within group)	*p* = 0.6434 (t-test)		*p* = 0.0419 (W)		*p* = 0.0528	*p* = 0.0866
Decrease in Apo B/Apo A1 ratio (n)	27		31			
Increase in Apo B/Apo A1 ratio (n)	21		18			
No change (n)	0		0			
p (chi-Square)	*p* = 0.619					

*T-test* Independent t-test, *t-test* Paired t test, *MW* Mann whitney test, *W* Wilcoxon signed rank test. *No change* Not included in the calculation

### Safety parameters

In the safety assessments, vital signs like systolic and diastolic blood pressure, heart rate, axillary temperature, respiratory rate were normal on the screening visits and during the study visits. No statistically significant changes in vital signs were observed between the groups. Routine urine parameters, hematology and biochemistry (SGOT, SGPT, serum bilirubin, serum creatinine, blood urea etc.) were also normal at baseline and throughout the study. The complete safety data is available as Additional file 6.

There were no withdrawals or drop outs reported and all 98 subjects completed the study with a good compliance to the study supplements. There were four adverse events reported in the study**.** One patient reported mild fever, one had mild headache and two patients reported mild gastritis. The site investigator classified these as mild in nature and having possible relationship with the product and resolved with routine medications. The un-blinding of the study groups revealed that three of the adverse events reported, were in patients belonging to the placebo group; the other one was amla extract group. Overall treatment compliance was extremely high with the Mean Value (MV = 87.5%) between both study arms (87.7% for amla extract and 87.3% for placebo).

## Discussion

The basic task of dealing with the epidemic of vascular diseases is primary prevention of dyslipidemia contributed by high blood cholesterol (TC) and triglycerides (TG) and associated lipoproteins. A single agent to treat the condition has not been available so far. Type 2 diabetes is associated with enhanced risk for CVD and ideally this condition also needs to be treated by the same agent. If such an agent is available, polypharmacy and associated adverse effects of consuming several drugs can be avoided. The results of the present study demonstrate the potential of a proprietary amla extract (TRI-LOW, Arjuna Natural Ltd., India) to adorn the role of such a single agent. Significant positive changes in individual lipid parameters have been achieved during a 12-week treatment regimen. However, the ability of the extract to treat hyperglycemia associated with T2DM needs to be reconfirmed since the only limited numbers of diabetics were included in the present study population.

Of the 49 participants of the amla group, 36 (73%) showed significant reduction in TC. A similar number showed reduction in LDL-C with several of them (26, 53%) achieving normal levels (< 100 mg/dl). Similary, 44 out of 49 participants achieved significant TG reduction. The extract had only marginal and insignificant effect on HDL-C levels. Results of VLDL-C levels were excellent with only 6 participants not responding to treatment. All except 8 participants achieved normal VLDL-C levels (< 30 mg/dl). Lipoprotein remnants, of which the most accessible is VLDL-C are TG-rich, increase CVD risk when TG levels are above 200 mg/dl [36], a criterion relevent to the present study population. Thus, the amla extract could favorably modulate all the significant lipid parameters. Results are in Table 2.

It was recognized early that results of single lipid parameters have limited success in risk prediction, and in efforts to improve CVD risk prediction, several lipid ratios have been defined. We employed two of the most useful of these ratios, namely, AIP and apoB/apoA-I. Both more or less predict the atherogenic versus antiatherogenic potential of a particular set of lipid parameters. AIP, expressed as log(TG/HDL (mmol)), has been proposed as a marker of plasma atherogenicity because the ratio is increased in people at higher risk CVD. AIP is also inversely correlated with LDL particle size, a parameter of high predictive value. Forty participants in the study (out of 49) recorded lower AIP values at the end of the study (Table 2).

The second ratio employed in the study is that of apoB and apo A-I. ApoB represents the atherogenic lipoprotein particles and apo A-I represent the anti-atherogenic ones. ApoB is present in VLDL, IDL, small dense LDL with one molecule of apoB in each of these atherogenic particles. Therefore, the total apoB reflects the total number of potentially atherogenic particles. ApoA-I is present in HDL particles and initiates the ‘reverse cholesterol transport’ by picking up excess cholesterol from peripheral cells and transfer it back to the liver in the HDL particles. In addition, apoA-I manifests anti-inflammatory and antioxidant effects. Thus, apoA-I reflects the athero-protective part of lipoprotein metabolism. The ratio of apoB/apoA-I, thus, reflects the balance of cholesterol transport in a simple way. Thirty one participants (63%) recorded lower apoB/apo A-I ratios (Table 2.). However, the decrease was not statistically significant.

The robust reduction observed in TG levels points to a potential role for the extract to treat diabetic dyslipidemia in particular wherein the importance shifts from cholesterol to triglycerides. In the Paris Prospective study, hypertriglyceridemia, and not hypercholesterolemia, predicted CVD mortality in a combined group of subjects with impaired glucose tolerance and diabetes [37]. Subjects with impaired glucose tolerance or diabetes at baseline (*n* = 943) were selected from the total population for a separate analysis of coronary heart disease mortality risk factors. After a follow-up of 11 years, in multivariate regression analysis using the Cox model, TG plasma level was the only factor positively and significantly associated with death from CVD (p less than 0.006). After a mean follow-up of 15 years, significant multivariate predictors of CVD death were plasma TG level, systolic blood pressure, and smoking. This epidemiological evidence of the consistency of hypertriglyceridemia as the most important predictor of CVD mortality in subjects with impaired glucose tolerance or diabetes suggests a possible role of hypertriglyceridemia in the excessive occurrence of atherosclerotic vascular disease in this category of patients.

Statins are the most employed lipid-lowering drugs used in clinical practice and are widely used to target LDL-C levels in primary and secondary cardiovascular prevention. Statins are generally very well tolerated but can cause swelling and tenderness in the muscles and in a very few cases lead to muscle damage. Independent of their hypolipidemic properties, statins interfere with events involved in bone formation and impede tumor cell growth. Other side effects include liver damage, increase in blood sugar and memory loss [38]. One of the serious drawbacks of statin therapy is the decrease in CoQ10 levels. In common with cholesterol, CoQ10 also uses mevalonate as its biosynthetic precursor. CoQ10 is an important factor in mitochondrial respiration. Primary and secondary deficiencies of CoQ10 result in a number of neurologic and myopathic syndromes. The association between treatment with statins and new-onset diabetes is of concern. Such an association has also been confirmed by some cohort studies and meta-analyses [39–42].

In February 2012, the Food and Drug Administration changed the safety label of statins to indicate the risk of increased HbA1c and fasting serum glucose levels [43]. Statins do not treat hypertriglyceridemia. Amla treatment caused only minor variations in CoQ10 levels (Table 2). This may indicate that amla inhibits some stage of cholesterol synthesis other than HMG CoA reductase. A second possibility is that the extract may facilitate increased LDL receptor expression.

Amla has been studied before [22–30] to treat dyslipidemia. But, the extract used in this study is qualitatively different from the earlier extracts wherein only the fleshy part of the amla fruits was used. The whole fruit (including a hard seed) has been used in preparing the extract used in the present study. Ninety eight participants were enrolled in the study and all completed the study. Treatment compliance for this study was extremely high with the Mean Value (MV = 87.5%) between both study arms (87.7% for amla extract and 87.3% for placebo). The very close mean values (MV), thus supporting the conclusion that compliance in both groups was similar. Furthermore, the lack of clinically significant abnormalities in physical findings in both groups, as observed on the screening and study visits as well as no withdrawals or drop outs, further supports the conclusion of good compliance by all 98 subjects. The four adverse events reported in the study were mild in nature and resolved with routine medications.

## Conclusions

A very significant reduction in the TC, TG, AIP and other lipid parameters strongly supports the efficacy of amla extract in patients at risk for CVD. Of note, the observed effects were noticed during a short span of only 12 weeks indicating its utility in the management of multiple components of dyslipidemia and cardiovascular health. An additional benefit of the use of amla extract over other standard of care therapies is the lack of change in serum CoQ10 levels. This suggests that amla extract may be a safer alternative to statins without severe adverse effects. A larger and longer term study is warranted to elucidate the mechanism of action of amla extract in dyslipidemic patients.

## Additional files

Additional file 1: Characterization of amla extract. (DOC 33 kb)

Additional file 2: Details of study sites. (DOCX 11 kb)

Additional file 3: Clinical study protocol. (PDF 343 kb)

Additional file 4: CONSORT Checklist. (DOC 219 kb)

Additional file 5: Full data of all visits. (DOCX 25 kb)

Additional file 6: Safety parameters. (DOCX 28 kb)

## Abbreviations

AIP: Atherogenic index of the plasma
Apo A1: Apolipoprotein A1
Apo B: Apolipoprotein B
CoQ10: Coenzyme Q10
CVD: Cardiovascular disease
HDL: High density lipoproteins
IDL: Intermediate density lipoproteins
LDL: Low density lipoproteins
T2DM: Type 2 diabetes mellitus
TC: Total cholesterol
TG: triglyceride
TSH: Thyroid stimulating hormone
VLDL: Very low density lipoproteins
