# Supplementary material for: A randomized, double blind, placebo controlled, multicenter clinical trial to assess the efficacy and safety of Emblica officinalis extract in patients with dyslipidemia
Source: BMC Complement Altern Med. 2019 Jan 22;19:27. doi: 10.1186/s12906-019-2430-y (PMC6341673; doi:10.1186/s12906-019-2430-y)
Supplement: Supplementary file 1 — Characterization of amla extract. (DOC 33 kb) [file 12906_2019_2430_MOESM1_ESM.doc]

**Reduction in Atherogenic Index of Plasma in Patients with Dyslipidemia: A Randomized, Double Blind, Placebo Controlled, Multicenter Clinical Trial with Amla Extract**

Haridas Upadya^1^, Prabhu S^2^, Aravinda Prasad^3^, Deepa Subramanian^4^, Swati Gupta^5*^ and Ajay Goel^6*^

^1^Aadhitya Adhikari Hospital, Contour Road, Gokulam, Mysore-570002, India

^2^ LifeCare Hospital, No.99, OM Complex, 20^th^ Main, Gangothri Circle, BTM 1^st^ Stage, Bangalore-560029, India

^3^Sri Venkateshwara Hospital, No.86, Hosur Main Road, Madiwala, Bangalore-560068, India

^4^Syncretic Clinical Research Services, No. 4, 5th cross, 11th Main Road, Vasanthnagar, Bangalore-560052, India (deepa@syncretic.in)

^5^Amrita School of Pharmacy, Amrita Institute of Medical Sciences and research centre, Amrita Vishwa Vidyapeetham, Amrita University, Kochi – 682041, India

^6^Center for Gastrointestinal Research; Center for Translational Genomics and Oncology, Baylor Scott & White Research Institute and Sammons Cancer Center, Baylor University Medical Center, Dallas, Texas, USA

**Supporting Information**

**CHARACTERIZATION OF AMLA EXTRACT**

Extraction and Standardization

10 kg of fresh amla fruits were extracted with ethyl acetate. The extract was filtered and concentrated under vacuum to get the crude extract. The crude extract was purified and standardized to contain about 35% polyphenols, 8% triterpenoids and 10% amla oil.

Quantification of polyphenols by UV spectroscopy

20 μg/mL reference standard solution of gallic acid was prepared in distilled water. 25 mg extract sample was suspended in distilled water and sonicated for 5 minutes. The suspension was filtered and volume was made up to 25 ml with distilled water. 2 mL each of sample and standard solutions were transferred in to 25 mL volumetric flask separately. 10 ml of distilled water and 1 mL of Folin-Ciocalteu reagent was added and the volume was made up with 10.75% anhydrous sodium carbonate (w/v) solution. After 30 min, the absorbance was measured at 760 nm and percentage of polyphenols was calculated as gallic acid equivalent.

Quantification of triterpenoids by UV spectroscopy

10 mg oleanolic acid reference standard was accurately weighed in to 10 mL volumetric flask and dissolved in ethyl acetate. The volume was made up to 10 mL with ethyl acetate to get 1 mg/mL concentration. Similarly, 10 mg of test sample was dissolved in ethyl acetate to get 1 mg/mL concentration of test solution. 500 mg of vanillin was dissolved in 10 mL of acetic acid to get vanillin-acetic acid solution.

2 mL of standard and sample solutions were taken into 10ml volumetric flask separately and evaporated to dryness. 0.5 mL of vanillin-acetic acid solution and 2 mL of perchloric acid was added to it. The resultant solution was heated at 65℃ for 20 min to allow color development and then cooled to room temperature. The solution was diluted with glacial acetic acid to the marked line and measured at 548 nm using a spectrophotometer.

Quantification of amla oil

Accurately weighed 10 gm extract sample was taken into an extraction thimble. The thimble was inserted into soxhlet extractor and extractor was connected to a 250 ml round bottom flask. The extraction was carried out with 150 ml of petroleum ether for 3 h on a water bath at 60^o^C. The petroleum ether was removed under vacuum and weight of oil was calculated.
