# Supplementary material for: A randomized, double blind, placebo controlled, multicenter clinical trial to assess the efficacy and safety of Emblica officinalis extract in patients with dyslipidemia
Source: BMC Complement Altern Med. 2019 Jan 22;19:27. doi: 10.1186/s12906-019-2430-y (PMC6341673; doi:10.1186/s12906-019-2430-y)
Supplement: Supplementary file 2 — Details of study sites. (DOCX 11 kb) [file 12906_2019_2430_MOESM2_ESM.docx]

Additional file 2

**Details of study sites**

| **S. No.** | **Name of the participating sites** |
| --- | --- |
| 1 | Aadhitya Adhikari Hospital, Contour Road, Gokulam, Mysore - 570002 |
| 2 | LifeCare Hospital, New No. 99 (Old No. 23/24), OM Complex, 20th Main, Gangothri Circle, BTM 1st Stage, Bangalore: 560029 |
| 3 | Sri Venkateshwara Hospital, No.86, Hosur Main Road, Madiwala,Bangalore: 560068 |
| 4 | Prashanth Hospital, Bommanahalli Circle, Hosur Main Road, Bangalore: 560068  Karnataka |
