# Supplementary material for: A randomized, double blind, placebo controlled, multicenter clinical trial to assess the efficacy and safety of Emblica officinalis extract in patients with dyslipidemia
Source: BMC Complement Altern Med. 2019 Jan 22;19:27. doi: 10.1186/s12906-019-2430-y (PMC6341673; doi:10.1186/s12906-019-2430-y)
Supplement: Supplementary file 3 — Clinical study protocol. (PDF 343 kb) [file 12906_2019_2430_MOESM3_ESM.pdf]

Clinical Study Protocol

**Protocol Title**	**:**	A Randomized, Double Blind, Placebo Controlled, Multicentre Clinical Trial to Assess the Efficacy and Safety of Amla Extract (AMX160) in Patients with Dyslipidaemia
**Project No.**	**:**	AN-03ASE 0115H2-SYN02
**Protocol Version No.**	**:**	01
**Protocol Date**	**:**	01-01-2015
**Superseded Version No.**	**:**	NA
**Superseded Version Date**	**:**	NA
**Test Products**	**:**	*Test*: Amla Extract (AMX160) of M/s Arjuna Natural Extracts Limited<br>*Reference*: Matching Placebo
**The Sponsor**	**:**	Arjuna Natural Extracts Ltd<br>P.B. No 126, Bank Road,<br>Alwaye, Kerala,<br>I N D I A -683 101.<br>Tel : +91 484 2532404, 4080400<br>Fax : +91 484 2622612<br>Email : lab@arjunanatural.com
**Clinical Laboratory**	**:**	Central Lab
**Centers**	**:**	Multicentric

This confidential document is the property of Arjuna Natural Extracts Ltd.
No unpublished information contained herein may be disclosed without prior written approval from Arjuna Natural Extracts Ltd.
Access to this document must be restricted to the relevant parties only.

STATEMENT OF COMPLIANCE

The trial will be conducted in accordance with the ethical principles that have their origin in the Declaration of Helsinki, and that are consistent with GCP and the applicable regulatory requirement(s).

The rights, safety and well-being of the trial subjects are the most important considerations and should prevail over interests of science and society.

A trial will be conducted in compliance with the protocol that has received prior institutional review board (IRB)/independent ethics committee (IEC) approval/favorable opinion.

Every individual involved in conducting a trial should be qualified by education, training, and experience to perform his or her respective task(s). Freely given informed consent will be obtained from every subject prior to clinical trial participation.

All clinical trials information will be recorded, handled, and stored in a way that allows its accurate reporting, interpretation and verification.

The confidentiality of records that could identify subjects will be protected, respecting the privacy and confidentiality rules in accordance with the applicable regulatory requirements(s).

Test products will be manufactured, handled, and stored in accordance with applicable good manufacturing practice (GMP). They will be used in accordance with approved protocol.

SPONSOR'S SIGNATURE PAGE

The signature below constitutes the approval of this protocol and the attachments, and provides the necessary assurances that this trial will be conducted according to all stipulations of the protocol, including all statements regarding confidentiality, and according to local legal and regulatory requirements and applicable regulations and ICH guidelines.

Protocol No. : AN-03ASE 0115H2-SYN02

TITLE: A Randomized, Double Blind, Placebo Controlled, Multicentre Clinical Trial to Assess the Efficacy and Safety of Amla Extract (AMX160) in Patients with Dyslipidaemia

Name of the Sponsor's Representative: **Dr. Benny Antony**____________

Signature of Sponsor's representative: ____________________________

Date: ____________________________

INVESTIGATOR'S SIGNATURE PAGE

The signature below constitutes the approval of this protocol and the attachments, and provides the necessary assurances that this trial will be conducted according to all stipulations of the protocol, including all statements regarding confidentiality, and according to local legal and regulatory requirements and applicable regulations and ICH guidelines.

Protocol No. AN-03ASE 0115H2-SYN02

TITLE: A Randomized, Double Blind, Placebo Controlled, Multicentre Clinical Trial to Assess the Efficacy and Safety of Amla Extract (AMX160) in Patients with Dyslipidaemia

Name of the Investigator: ____

Investigator's Signature: ____

Date: ____

Abbreviations

Sr. No.	Abbreviation	Full Form
1.	AE	Adverse Event
2.	AIP	Atherogenic Index of Plasma
3.	ApoA1	Apo lipoprotein A1
4.	ApoB	Apo lipoprotein B
5.	CAD	Coronary Artery Disease
6.	CHD	Coronary Heart Disease
7.	CRA	Clinical Research Associate
8.	CRF	Case Record Form
9.	EC	Ethics Committee
10.	FBS	Fasting Blood Sugar
11.	GCP	Good Clinical Practise
12.	HbA1c	Glycosylated Haemoglobin
13.	HDL	High Density Lipoproteins
14.	HMG Co-A	3-hydroxy-3-methylglutaryl-coenzyme A
15.	ICF	Informed Consent Form
16.	IEC	Institutional Ethics Committee
17.	IRB	Institutional Review Board
18.	LAR	Legally Acceptable Representative
19.	LDL	Low Density Lipoproteins
20.	mg	Milligram
21.	mITT	Modified Intention to Treat
22.	PP	Per Protocol
23.	QA	Quality Assurance
24.	SAE	Serious Adverse Event
25.	SD / SE	Standard Deviation / Standard Error
26.	TC	Total Cholesterol
27.	TG	Triglycerides
28.	PIS	Patient Information Sheet
29.	Urine R/M	Urine Routine and Micro test
30.	VLDL	Very Low Density Lipoproteins

Table of Contents

Abbreviations **5**
1 Background and Introduction **8**
2 Study Rationale **9**
3 Study Objective **9**
4 Study Design **9**
4.1 Overview of the study design 9
4.1.1 Type of study 9
4.1.2 Study groups 10
4.1.3 Number of study subjects and subject number assignment 10
4.1.4 Type, sequence and duration of study periods 10
4.2 Flow chart of the study 11
4.3 Brief description of the methods and procedures 11
4.4 Brief discussion of study design 12
5 Study Population **12**
6 Subject Eligibility **12**
6.1 Inclusion Criteria 12
6.2 Exclusion Criteria 13
6.3 Discontinued subjects 13
7 Study Assessments **14**
7.1 Assessment of Efficacy 14
7.2 Assessment of Safety 16
7.3 Lab Investigations 17
8 Study Conduct **18**
8.1 Protocol violation, deviation, waiver or amendments 21
9 Study Treatment **22**
9.1 Dosing schedule 22
9.2 Supply and administration of Test Product 22
9.3 Dose modification for Test Product toxicity 22
9.4 Assessment of compliance for dosing 22
9.5 Possible drug interactions 23
9.6 Concomitant therapy 23
9.7 Blinding procedures 23
9.8 Unblinding procedures 23
10 Adverse Events **23**
10.1 Description of adverse events 23
10.2 Procedures to evaluate an adverse event 24
10.2.1 Definitions 24
10.2.2 Adverse Events Recording 24
11 Ethical Considerations **27**
11.1 Good Clinical Practice (GCP) 27
11.2 Risk / benefit assessment 27

11.3 Ethics Committee review and communications 27
11.4 Informed consent process 28
11.5 Statement of subject confidentiality (including ownership of data and coding procedures) 28
11.6 Financial considerations for the subjects 29
12 Study Monitoring and Supervision 29
12.1 Study monitoring 29
12.2 Study materials 29
12.3 Filling of Case Report Forms 29
12.4 Data handling & record keeping 30
12.5 Source data accessibility 30
12.6 Data entry and verification 30
12.7 Archiving 30
12.8 Termination of study 31
13 Test Product Management 31
13.1 Test Product description and packaging 31
13.2 The precise dosing regimen 31
13.3 Method of packaging, labelling and blinding of Test Product 31
13.4 Method of treatment assignment and subject identification coding 32
13.5 Storage conditions for Test Product 32
13.6 Test Product accountability 32
13.7 Procedures for handling unused Test Products 32
14 Data Analysis 32
14.1 Sample size calculation 33
14.2 Data Entry and Data Validation 33
14.3 Missing, Unused or Spurious Data 33
14.4 Statistical analysis 33
15 Financial Considerations 34
16 Publication Policy 34
17 Sponsor's Representative / Medical Expert 34
18 Appendices 34
18.1 World Medical Association - Declaration of Helsinki 35
18.2 Randomization Schedule 40
18.3 Serious Adverse Event (SAE) Reporting Form 42
18.4 Unscheduled Visit Form 43
18.5 Overall Tolerability Evaluation 44
19. Bibliography 45

1 Background and Introduction

Dyslipidaemia is one of the most frequently implicated risk factors for development of atherosclerosis.[1] A strong association exists between hyperlipidaemia and coronary artery disease (CAD), cerebrovascular stroke, and peripheral vascular disease. In the peripheral circulation, it can cause intermittent claudication and gangrene and can jeopardize limb viability. The hypolipidaemic medications have attracted considerable attention because of their potential to prevent cardiovascular disease by retarding the accelerated atherosclerosis in hyperlipidaemic individuals. The medications used in the management of hyperlipidaemia are bile acid sequestrants, nicotinic acid, fibric acid derivatives, and 3‑hydroxy‑3‑methylglutaryl‑coenzyme A (HMG Co‑A) reductase inhibitors.[2] Although all these medications are effective in the management of dyslipidaemia, they are associated with significant side effects like hepatitis, rhabdomyolysis, myopathy, etc. and thus search for safer alternatives continues.

Various medicinal plants used in the traditional systems of medicine have shown immense potential in management of dyslipidaemia and CAD without major adverse events. *Emblica officinalis* (Amla or Indian gooseberry) is one such medicinal plant which has been shown to be effective in management of dyslipidaemia in experimental animals and in pilot clinical studies without major side effects.

In vitro and animal studies have shown that the flavonoids from *E. officinalis* effectively reduce lipid levels in serum and tissues and exert a significant inhibitory effect of hepatic HMG CoA reductase enzyme activity.[3] Previous studies in rabbits have confirmed that Amla Extract of M/s Arjuna Natural Extracts Limited in effective management of dyslipidaemia by reducing Low Density Lipoproteins (LDL) and increasing the High Density Lipoproteins (HDL) levels.[4] Beside the effect on serum lipids, Amla Extract is also known to have potent anti-oxidant effects as established in several test systems such as superoxide radicals, lipid peroxide formation induction by Fe+++/ADP ascorbate system, hydroxyl radical scavenging action and in systemic augmentation of antioxidant enzymes in the brain of laboratory animals.[5-8]

A few pilot clinical studies have also shown the beneficial effects of Amla Extract in patients with dyslipidaemia. In a non-comparative clinical study, Amla Extract of M/s Arjuna Natural Extract Limited at doses of 500 mg/day and 1000 mg/day significantly reduced the levels of risk factors of CAD arising from dyslipidaemia and inflammation.[9] In another placebo controlled trial, Amla Extract of M/s Arjuna Natural Extract Limited at a dose of 500 mg twice daily, showed a significant reduction in Total Cholesterol (TC), LDL, Very Low Density Lipoproteins (VLDL) and Triglycerides (TG) and a significant increase in serum HDL as compared to placebo over a period of 4 months.[10] Another clinical study comparing the efficacy and safety of Amla Extract (500 mg/day) with Simvastatin (20 mg/day) has not shown any difference in the beneficial effects of the two active treatments on TC, LDL and HDL.[11]

This clinical study is being planned to assess the efficacy and safety of Amla Extract of M/s Arjuna Natural Extracts Limited, Kerala for management of dyslipidaemia. Beside the conventional lipid parameters like TG, TC, LDL, and HDL various other parameters have shown correlation with progression of coronary artery disease. The most important of these parameters include the Atherogenic Index of Plasma (AIP), Apo lipoproteins A1, Apo lipoprotein B and their ratio, Homocysteine levels, Coenzyme Q10 and chronic inflammatory markers like hs-CRP.[12-16] This study is being planned to assess the effect of Amla Extract of M/s Arjuna Natural Extracts Limited, Kerala on these parameters besides the effect on the conventional lipid parameters. Moreover, as Amla Extract is known to have an effect on blood sugar levels[17], HbA1c and fasting glucose shall also be measured during the study.

2 Study Rationale

Randomized, double blind, placebo controlled studies are considered to be of the highest standards for the assessment of efficacy and safety of a study medication; accordingly the same design is used for this study. Patients with dyslipidaemia will be enrolled in the study to assess the effect of Amla Extract (AMX160) on lipid levels. Patients with severe disease or having 2 or more risk factors as per the ATP III guidelines will not be enrolled in the study as this is a placebo controlled trial and half of the patients in the study will not be receiving any active treatment.[18] As most of the lipid lowering agents provide a peak action by 4 weeks and also various international clinical studies evaluating the efficacy and safety of medications for dyslipidaemia have a 12 week study period, a follow up of 12 weeks is planned in the study. Moreover, long term study cannot be justified with a placebo arm. All the patients enrolled in the study will also be asked to initiate lifestyle changes (healthy diet with aerobic exercise at least 4 days a week) along with the study medication.

3 Study Objective

The objective of this clinical study is to evaluate the efficacy and safety of Amla Extract (AMX160) of M/s Arjuna Natural Extracts Limited, Kerala as compared to the matching placebo in patients with dyslipidaemia aged 30-65 years.

4 Study Design

4.1 Overview of the study design

4.1.1 Type of study

A prospective, randomized, double blind, parallel, placebo controlled, multicentre, clinical study

4.1.2 Study groups

Test group: Amla Extract (AMX160) 500 mg capsule of M/s Arjuna Natural Extracts Limited, Kerala

Reference group: Matching Placebo

4.1.3 Number of study subjects and subject number assignment

A total of 98 patients at multiple centres in India will be simultaneously enrolled in this clinical trial to compare the efficacy and safety of the test versus reference product (refer section 14.1 for details of sample size calculation). The enrolled patients would be assigned to either of the 2 study groups according to the centralized computer generated randomization in a 1:1 ratio.

4.1.4 Type, sequence and duration of study periods

This is a parallel treatment group study and all the enrolled patients will be instructed to take study medication for a treatment period of 12 weeks. There will be a total of 5 visits: Screening, Randomization (Day 0), Follow up (Week 4), Follow up (Week 8) and End of study visit (Week 12). Laboratory investigation of all the efficacy parameter will be conducted at screening, on all follow up visits and end of study while laboratory investigations for safety will be carried out on screening and end of the study.

4.2 Flow chart of the study

Visit 1 (Screening, Day -7)
Written Informed Consent
Check for eligibility criteria
Clinical Examination; Concomitant diseases / Medication
Laboratory Investigations (Efficacy and Safety); ECG

⇓

Visit 2 (Randomization, Day 0)
Re-check for eligibility criteria
Amla Extract (AMX160) or Placebo (500 mg capsule BD)
Clinical Examination; Concomitant diseases / Medication
Laboratory Investigations (No Blood Collection)

⇓

Visit 3 (Follow up, Day 28 ± 5 Days)
Clinical Examination; Concomitant diseases / Medication
Laboratory Investigations (Efficacy)

⇓

Visit 4 (Follow up, Day 56 ± 5 Days)
Clinical Examination; Concomitant diseases / Medication
Laboratory Investigations (Efficacy)

⇓

Visit 5 (End of Study, Day 84 ± 5 Days)
Clinical Examination; Concomitant diseases / Medication
Laboratory Investigations (Efficacy and Safety); ECG

4.3 Brief description of the methods and procedures

Patients with dyslipidaemia aged 30-65 years will be evaluated as per the Inclusion & Exclusion Criteria as mentioned below (section 6) and will undergo a thorough physical (general and systemic) examination and appropriate laboratory investigations (mentioned in section 7.3) to rule out other concomitant diseases during the screening visit. Eligible patients will receive either Amla Extract (AMX160) 500 mg capsule of M/s Arjuna Natural Extracts Limited, Kerala or a matching placebo capsule twice daily according to a centralized computer generated randomization plan. This would be a double blind study and hence, neither the investigator nor the patients will know which medication is being dispensed to him. All the patients enrolled in the study will also be asked to initiate lifestyle changes (healthy diet with exercise at least 4 days a week) along with the study medication.

After randomization, patients will be followed up on outpatient basis for period of 84 days (12 weeks) with scheduled visits on day 28, day 56 and day 84 post-randomization. Efficacy assessments will be carried out by laboratory investigations on each of the scheduled visits (except randomization visit). Clinical adverse events monitoring would be done on each visit and complete laboratory check-ups (both haematological and biochemical) and ECG will be repeated at the end of the study (12 weeks) so as to assess the safety of the study medication. Laboratory values obtained during the screening visit will be considered as baseline values for efficacy and safety analysis. Though every effort will be made to adhere to the schedule of visits, a window period of ± 5 days will be permitted for all the visits.

4.4 Brief discussion of study design

This will be a randomized, double blind, parallel, placebo controlled, multicentre study to evaluate the efficacy and safety of Amla Extract (AMX160) 500 mg capsule of M/s Arjuna Extract Limited, Kerala compared to a placebo capsule in patients with dyslipidaemia. Randomization prevents selection / treatment allocation bias and blinding will prevent the bias in the assessment of efficacy and safety (adverse events) due to the study medication. Randomized patients will be simultaneously enrolled in either study group as per their randomization number and will be followed up in a parallel manner till the completion of study.

5 Study Population

Patients with dyslipidaemia aged 30-65 years will be enrolled in this study. A total of 98 patients at multiple centres will be simultaneously enrolled in this clinical study. Enrolment of patients at study sites would be competitive i.e. if enrolment is slow or inadequate at one site, then more number of patients may be enrolled at other sites in order to complete the study

6 Subject Eligibility

6.1 Inclusion Criteria

1. Patients aged 30-65 years
2. Patients with dyslipidaemia having Triglycerides (TG) >200 mg/dL, LDL cholesterol > 130 mg/dL, Total cholesterol > 200 mg/dL and HDL cholesterol < 40 mg/dL for men and < 50mg/dL for women
3. Patients not taking any medication (including herbal product) for management of dyslipidaemia since last 4 weeks
4. Informed consent of the patient

6.2 Exclusion Criteria

1. Patients with more than 2 of the following risk factors -
   - Cigarette smoking
   - Hypertension (BP >140/90 mmHg or on antihypertensive medication)
   - Family history of premature CHD (CHD in male first degree relative <55 years; CHD in female first degree relative <65 years)
   - Age (men >45 years; women >55 years)
2. Patients with uncontrolled cardiovascular disease or advanced atherosclerosis (e.g. history of stroke, myocardial infarction, life-threatening arrhythmia, or coronary revascularization within the preceding 6 months; unstable angina; congestive heart failure; known or suspected clinically significant valvular heart disease or uncontrolled hypertension (>160/100 mm of Hg or use of antihypertensive medications, dose of which is not stable in the last one month))
3. Patients with very high triglyceride levels i.e. > 500 mg/dL
4. Patients with FBS more than 150 mg/dL, using insulin, glitazones. Patients using other hypoglycaemics, dose of which is not stable in last one month
5. Pregnancy, lactation and female patients not using acceptable contraceptive measures (double barrier methods, oral or injectable hormonal contraceptives or surgical sterilization)
6. Patients with hepatic impairment (SGOT or SGPT levels > 3 Upper Limit of Normal (ULN)) or renal impairment (serum creatinine ≥ 2.0 mg/dl)
7. Patients with any other severe systemic illness and in the opinion of the investigator would be noncompliant with the visit schedule or study procedures
8. Patients with known history of hypersensitivity to amla or any product containing amla extract
9. Patients with continuing history of alcohol and / or drug abuse.
10. Patients with any other serious concurrent illness or malignancy.
11. Participation in another clinical trial in the past 3 months

6.3 Discontinued subjects

The investigator may withdraw a subject from the study if he/she meets any of the following withdrawal criteria:

1. Any patients / patient’s legally acceptable representative who wishes to withdraw his / her consent for participation in the study
2. The subject suffers from significant intercurrent illness or undergoes surgery during the course of the study.
3. Any patient who requires/uses an unacceptable concomitant medication as per the Protocol
4. Failure to take 80% of the study medication during the study period

5. If it is felt in Investigator's / Medical Expert's opinion that it is not in the patients' best interest to continue.

The date the subject is withdrawn from the study and the reason thereof will be documented in the CRF. Withdrawn subjects will be considered for modified intention to treat efficacy analysis, if they have completed at least 1 visit post randomization (visit 3; week 4) with laboratory evaluation. Though every effort will be made to adhere to the schedule of visits, a window period of ± 5 days will be permitted for all the visits

7 Study Assessments

All the subjects will undergo a thorough general physical examination including recording of vitals such as temperature, pulse, respiratory rate; systemic examination of cardiovascular, respiratory, central nervous systems and abdomen. The subjects will be evaluated as per the Inclusion & Exclusion Criteria. Haematological and biochemical laboratory investigations will be carried out as mentioned in section 7.3. If the patient is suffering from any other concomitant diseases and taking medications for the same, these details will also be recorded in the CRF.

7.1 Assessment of Efficacy

Lipid Parameters

Efficacy of the study medication will be assessed by serum lipid parameters including Triglycerides, Total Cholesterol, LDL and VLDL. All the lipid parameters will be measured after at least 8 hours of fasting.

Atherogenic index of Plasma[12]

The atherogenic index of plasma (AIP) is defined as the base 10 logarithm of the ratio of the concentration of triglyceride (TG) to high density lipoprotein cholesterol (HDL-C), where each concentration is expressed in mmol/L.

$$\text{AIP} = \log_{10}\left(\frac{\text{TG}}{\text{HDL-C}}\right)$$

This quantity has been shown to reflect the distribution of particle sizes in lipoprotein subclasses and correlates significantly with the presence of risk factors for atherosclerosis such as gender, age, dyslipidemia and diabetes as well as positive findings on coronary angiography. Based on epidemiological data three risk categories for AIP have been suggested: low < 0.11, intermediate: 0.11–0.21 and high > 0.21

Apo lipoprotein B (ApoB) and Apo lipoprotein A1 (ApoA1)[13]

Apo lipoproteins (apo) AI and B are structural proteins for high density lipoproteins (HDL-C), and the very low density-low density lipoprotein spectrum (VLDL-LDL) respectively. There is mounting evidence that implicates Apo lipoprotein B and Apo lipoprotein AI levels in the pathogenesis of CAD. This study will measure the levels of Apo lipoproteins (Apo) AI and B and their ratio (ApoA1/ApoB) during the course of the study.

FPG and HbA1c[17]

Amla extract is known to have an effect on blood glucose levels therefore, fasting plasma glucose and HbA1c will also be measured during the study. HbA1c will be measured by a method that is certified by the National Glycohemoglobin Standardization Program. For FPG, patient will be fasted, defined as no caloric intake for at least 8 hours.

Homocysteine levels[14]

High levels of plasma homocysteine are associated with increasing risk of damage to the arterial endothelium and thrombus formation.

Coenzyme Q10[15]

Coenzyme Q10 is an intracellular antioxidant that protects the membrane phospholipids, mitochondrial membrane protein, and LDLC from free radical-induced oxidative damage. It has been demonstrated that coenzyme Q10 has a cardio-protective impact on CAD. A higher level of plasma coenzyme Q10≥(0.52 µmol/ L) is significantly associated with a reduced risk of CAD.

hs-CRP[16]

C-reactive protein (CRP) is a marker for inflammation and its increasing levels indicate an increased state of inflammation in the body. The levels are correlated with increasing risk of CAD. High Sensitivity-CRP levels Less than 1.0 mg/L are associated with ‘Low’ risk, 1.0-2.9 mg/L are associated with ‘Intermediate’ risk and levels Greater than 3.0 mg/L are associated with ‘High’ risk.

ATP goals

Proportion of patients achieving all the three ATP goals i.e. Triglyceride < 150 mg/dL, LDL < 100 mg/dL, HDL > 40 will be calculated for the two groups.

HMG Co-enzyme Reductase Activity[19]

HMG-CoA reductase is the rate-controlling enzyme of the mevalonate pathway, the metabolic pathway that produces cholesterol and other isoprenoids. Medications affecting cholesterol synthesis inhibit the activity of this enzyme. The activity of HMG-CoA reductase will be measured in the two groups at baseline and end of the study using HMGR activity kit (----Details of Kit to be added----). This will help us understand the probable mechanism of action of Amla Extract in reducing lipid levels.

The primary efficacy endpoint would be:

1. Mean percent change in TG levels at the end of 12 weeks in the two groups

The secondary efficacy endpoint would be:

1. Mean percent change in Total cholesterol, LDL and VLDL levels at the end of 12 weeks in the two groups
2. Mean change in the Atherogenic Index of Plasma (Log (TG/HDL))
3. Mean change in apolipoprotein B (apoB), apolipoprotein A1 (apoA1) and their ratio at the end of 12 weeks in the two groups
4. Mean change in FPG and HbA1c levels at the end of 12 weeks in the two groups
5. Mean change in hs-CRP, homocysteine, Coenzyme Q10 levels at the end of 12 weeks in the two groups
6. Proportion of patients achieving ATP goals (Triglyceride < 150 mg/dL, LDL< 100 mg/dL, HDL> 40) at the end of the study
7. Change in the HMG-CoA reductase activity at the end of the study in the two groups

7.2 Assessment of Safety

The safety of the study medication will be assessed by recording the adverse events occurring during the entire course of the study, as mentioned in detail in “Adverse Events” section of the protocol. All observed (during general and physical examination of patients or in the laboratory parameters) or volunteered adverse events regardless of suspected causal relationship to the study test product will be recorded on the adverse event page of the CRF. ECG of all the patients will be done at the screening and at the end of the study.

12 Lead Electrocardiogram[20]

A standard 12 lead ECG will be recorded at the screening and at the end of the study as per the methodology approved by the British Cardiovascular Society for recording a standard 12 lead electrocardiogram. Both absolute QT interval and corrected QT interval will be noted from precordial leads and Lead II. QT interval will be corrected using Fridericia's formula ($QTc = QT/RR^{0.33}$ i.e. Observed QT interval divided by cube root of RR interval, in seconds).

Overall tolerability evaluation:

The overall tolerability to study medication will be evaluated at the end of study as per the following criteria:

Grade	Description
Excellent	No adverse event
Good	Mild adverse event(s) reported which subsided with or without medication and did not necessitate stoppage of study medication
Fair	Moderate adverse event(s) reported which subsided with or without medication and did not necessitate stoppage of study medication
Poor	Severe or serious adverse event(s) which necessitated stoppage of study medication

The safety endpoints would be:

- Local and systemic adverse events reported during the study
- Serious adverse events reported during the study
- Overall tolerability evaluation at the end of study

7.3 Laboratory Investigations

A Central Clinical Pathology Laboratory will be used for all laboratory investigations. Methods to be used for the laboratory tests are specific to the Laboratory. Lab investigations to be conducted are; Haematological parameters include haemoglobin levels, total leucocyte count, Absolute Neutrophil Count (ANC), platelet count. Biochemical parameters include fasting blood sugar, blood urea, serum creatinine, serum bilirubin, SGOT, SGPT, TG, TC, LDL, HDL, VLDL, Apo A1, Apo B, hs-CRP, Homocysteine, HbA1C, TSH, CoQ10 and HMG Coenzyme Reductase activity. Serum pregnancy test will be done during screening and end of study in females of child bearing potential.

The schedule for laboratory test is shown in table below:

Parameter	Visit 1 Screening)	Visit 2 (Day 0)*	Visit 3 (Week 4)	Visit 4 (Week 8)	Visit 5 (Week 12)
**Efficacy**					
**TG**	√		√	√	√
**LDL**	√		√	√	√
**HDL**	√		√	√	√
**VLDL**	√		√	√	√
**TC**	√		√	√	√
**Apo A1**		√			√
**Apo B**		√			√
**hs CRP**		√			√
**Homocysteine**		√			√
**FPG**	√		√	√	√
**CoQ10**		√			√
**HbA1c**		√			√
**HMG Coenzyme Reductase activity**		√			√
**Safety**					
**CBC**	√				√
**LFT (SGOT, SGPT, bilirubin)**	√				√
**RFT (Urea, creatinine)**	√				√
**TSH**		√			√
**SPT**	√				√
**Urine (R/M)**	√				√
**ECG**	√				√
* Blood sample will not be collected; analysis to be carried out using blood collected during screening visit					

8 Study Conduct

The patients fulfilling the inclusion and exclusion criteria will be enrolled in the trial and will be randomized to receive either of the study medications in a double blinded

manner. The patients will be followed on an outpatient basis as per the schedule of visits mentioned below. Though every effort will be made to adhere to the schedule of visits, a window period of ± 5 days for will be permitted. Further, on all the scheduled visits the following assessments will be done and recorded in the CRF.

Activity Chart

Activity	Visit 1 (Day -7)	Visit 2 (Day 0)	Visit 3 (Day 28 ± 5 days)	Visit 4 (Day 56 ± 5 days)	Visit 5 (Day 84 ± 5 days)
Informed Consent	√				
Demographics & Medical History	√				
Inclusion / Exclusion criteria check	√	√			
ECG	√				√
Lab investigations	√	√*	√	√	√
Vital Signs & Clinical Examination	√	√	√	√	√
Dispensing of Study Medication		√	√	√	
Study Medication Count			√	√	√
Recording of Adverse Events			√	√	√
Concomitant Disease and Medications	√	√	√	√	√
Overall Assessment of Tolerability					√
Study Completion					√

*No blood collection. Use blood sample collected on screening visit

Visit 1: Screening (Upto Day-7)

- Written informed consent of the patient
- Demographic details & medical history
- General physical and systemic examination
- Concomitant diseases and medications

- Inclusion and exclusion criteria check
- Blood collection for laboratory tests (only TC, TG, LDL, HDL, VLDL, FPG, CBC, LFT, RFT, SPT will be analysed first; blood sample will be stored for further analysis); Urine R/M; ECG
- SPT in females of child bearing potential

Visit 2: Randomization (Day 0)

- Inclusion & Exclusion Criteria re-check
- General physical and systemic examination
- Laboratory investigations - If patient satisfies all inclusion and exclusion criteria, other investigations i.e. Apo A1, Apo B, hs CRP, TSH, Homocysteine CoQ10, HbA1c and HMG Coenzyme Reductase activity will be done utilizing the same blood sample as collected at time of screening. No fresh blood sample will be collected on this visit
- Randomization and dispensing of study medication
- Recording of concomitant diseases and medications
- Instructions for lifestyle modification (healthy diet with aerobic exercise at least 4 days a week)

Visit 3: Follow-up visit (Day 28 ± 5)

- General physical and systemic examination
- Study Medication count
- Adverse event recording
- Concomitant diseases and medications
- Blood sample collection for laboratory analysis
- Dispensing Study Medication
- Instructions for lifestyle modification (healthy diet with aerobic exercise at least 4 days a week)

Visit 4: Follow-up visit (Day 56 ± 5)

- General physical and systemic examination
- Study Medication count
- Adverse event recording
- Concomitant diseases and medications
- Blood sample collection for laboratory analysis
- Dispensing Study Medication
- Instructions for lifestyle modification (healthy diet with aerobic exercise at least 4 days a week)

Visit 5: End of Study visit (Day 84 ± 5)

- General physical and systemic examination
- Study Medication count
- Adverse event recording
- Concomitant diseases and medications
- Blood collection for laboratory investigations and SPT in females of child bearing potential, Urine R/M, ECG
- Overall tolerability evaluation

Unscheduled Visits

Unscheduled visits will be allowed any time during the study for assessment and management of AEs and any concurrent clinical conditions and the same shall be captured in the Unscheduled visit form.

8.1 Protocol violation, deviation, waiver or amendments

During this study examples of deviations may include, but are not limited to:

a) Participant seen outside window period (± 5 days)
b) Missed or incomplete study procedure (e.g. examination)

During this study following will be considered as protocol violations:

a) Failure to report within ± 7 days
b) Failure to obtain written informed consent
c) Missing Informed Consent Form (ICF)
d) ICF does not contain all the required signatures
e) ICF used was not the current EC approved version
f) Participant did not meet eligibility criterion
g) Prescribed dosing outside protocol guidelines
h) A breach of confidentiality
i) Falsifying research or medical records
j) Participant receives wrong treatment
k) Randomization of participant prior to EC approval of protocol
l) Subject withdrawal criteria not followed
m) Use of prohibited concomitant treatments

Any deviation or violation from the Protocol should be duly explained with justification(s) thereof by the concerned investigator. The Investigator should promptly report to the Ethics Committee, sponsor's representative / medical expert, deviation / violation from the Protocol, if any which may cause immediate hazards to the subjects. There will be no protocol waiver in this study. Subjects dropped out before the study completion will not be replaced. Patients violating the Protocol will

not be included in the Per Protocol efficacy analysis while all the patients (including those with deviations and violations) will be considered for modified Intention To Treat efficacy analysis if they have completed visit 3 (week 4) with laboratory evaluation. All the subjects who have been dispensed study medication and have completed visit 3 (week 4) will be considered for safety evaluation.

Neither the investigator nor Arjuna Natural Extracts Limited will alter this study protocol. Amendments should be made only in exceptional cases once the study has started. Any change in the protocol should be approved by IEC / IRB, wherever necessary before implementation.

9 Study Treatment

9.1 Dosing schedule

Patients will be instructed to take one capsule of Amla Extract (AMX160) 500mg of M/s Arjuna Natural Extract Limited Kerala or matching placebo twice daily (morning and evening) after meals.

9.2 Supply and administration of Test Product

The study medications will be labelled and supplied by M/s Arjuna Natural Limited, Kerala. The study medications will be manufactured following all the relevant regulations. Matching placebo capsules will be used as reference product. All supplies will be stored in accordance with the manufacturer’s instructions separately from normal hospital / practice stocks. The study medications will be provided in a total of 3 containers, each containing 66 capsules of either Amla Extract (AMX160) or placebo. (56 capsules will be used during 28 day period and 10 extra capsules will be given for 5 day buffer period during each visit). The patients will be given one container of study medication during visit 2 (Day 0), 3 (Day 28) and 4 (Day 56).

9.3 Dose modification for Test Product toxicity

No dose modification of the study medication will be allowed during the study.

9.4 Assessment of compliance for dosing

The patients will be asked to bring the container of the study medication on every follow-up visit and compliance for dosing will be assessed by medication count by study personnel out of the sight of the patient.

9.5 Possible drug interactions

This is a food supplement. No drug interactions are known for this product.

9.6 Concomitant therapy

Patients will not be permitted to take any medication (allopathic or herbal preparation) affecting lipid levels in a concomitant manner. Further, the concomitant use of medications for the treatment of other concomitant diseases, not known to interact with study medication would be permitted as deemed necessary by the investigator. A list of all the concomitant medications taken by the patient would be recorded in the Case Report Form (CRF).

9.7 Blinding procedures

This is a double-blind study. To maintain blindness of study treatments, placebo capsules resembling in every manner to Amla Extract (AMX160) 500mg Capsule will be used. Both the study medications will be coded centrally with randomization numbers as per the computer generated block randomization schedule. Sealed envelopes / scratch cards will be provided to the investigators by the sponsor (one each for each randomization number) indicating the randomization number and the test product contained therein. The sealed envelopes / scratch cards will be kept by the investigator in a safe but accessible place should the need for breaking the blinding medication code arises in an emergency.

9.8 Unblinding procedures

The sealed envelope / scratch cards containing the test product details (randomization number and study test product name) may be opened only if knowledge of the treatment regimen can influence or assist with medical management of the patient in an acute emergency. Every effort must be made to contact the sponsor before breaking the blinding code. If it is not possible to contact the sponsor and the situation is an emergency, the investigator may break the blinding code and contact the sponsor as soon as possible. The date, time and reason for breaking the code will be documented in the CRF. Patient will be discontinued from the trial once the blinding code is broken. Opened as well as unopened code envelopes / scratch cards should be returned to the sponsor at the end of the study.

10 Adverse Events

10.1 Description of adverse events

Amla Extract (AMX160) is an extract of Amla fruits and is well tolerated. No adverse events have been reported with the use of the product till now in various clinical

studies conducted earlier in patients with dyslipidaemia. Patients using Amla Extract may develop abdominal pain, nausea, fever or vomiting.[21]

10.2 Procedures to evaluate an adverse event

10.2.1 Definitions

Adverse Event following immunization (AEFI): Any untoward medical occurrence which follows usage of study medication and which does not necessarily have a causal relationship with the usage of the medication. The adverse event may be any unfavourable or unintended sign, abnormal laboratory finding, symptom or disease.

Serious Adverse Events (SAEs):

Any untoward medical occurrence that at any dose:

1. Results in death,
2. Is life-threatening,
3. Requires inpatient hospitalization or prolongation of existing hospitalization,
4. Results in persistent or significant disability/incapacity
5. Is a congenital anomaly/birth defect, or
6. Requires intervention to prevent permanent impairment or damage

10.2.2 Adverse Events Recording

Adverse events will be elicited using the open question - Have you noticed any change in your health since your last visit? Events involving adverse drug reactions, illnesses with onset during the study or exacerbation of pre-existing illnesses should be recorded.

All observed or volunteered adverse events regardless of treatment group or suspected causal relationship to the study test products will be recorded on the adverse event page of the CRF. Safety data including adverse events will be tabulated for clinical review.

For all adverse events, the Investigator must pursue and obtain information adequate both to determine the outcome of the adverse event and to assess whether it meets the criteria for classification of a serious adverse event requiring immediate notification to the Sponsor’s representative / medical expert.

Follow-up of the adverse event, even after the date of therapy discontinuation, is required if the adverse event or its sequel persist. Follow-up is required until the event or its sequel resolve or stabilises at a level acceptable to the Investigator and the study monitor.

All the adverse events will be documented in the respective CRFs including

1. Type of adverse event
2. Date of onset
3. Severity
4. Treatment given
5. Association or relationship to the study medication
6. Date of resolution
7. Duration
8. Outcome
9. Whether the adverse event is serious or not

The SAE will be recorded in an additional 'Serious Adverse Event Reporting Form'. The SAE will be reported to the Sponsor's representative / medical expert and to the Ethics Committee that has given approval to the study Protocol as per the applicable guidelines.

The Principal Investigator must report all serious and unexpected adverse events to the Sponsor's representative / medical expert and to the Ethics Committee that has given approval to the study Protocol at that centre within 24 hours of the occurrence. The report of SAE of death, after due analysis shall be forwarded by the Investigator to the Chairman of Ethics Committee and the Head of the Institution where the trial has been conducted within 10 calendar days of occurrence of SAE of death. The report of SAE other than death, after due analysis shall be forwarded to the Chairman of Ethics Committee and the Head of the Institution where the trial has been conducted within 10 calendar days of occurrence of SAE.

Sponsor's representative / medical expert will forward report of SAE of death occurring in the clinical trial, after due analysis to the Chairman of the Ethics Committee and the Head of the Institution where the clinical trial has been conducted within 10 calendar days of occurrence of the SAE of death. The report of the SAE other than death, after due analysis, will be forwarded by the Sponsor's representative / medical expert to the Chairman of the Ethics Committee and the Head of the Institution where the clinical trial has been conducted within 10 calendar days of occurrence of the SAE other than death. Sponsor's representative / medical expert will also communicate any unexpected SAE occurring during the clinical trial to other Investigator(s) participating in the study.

For all serious adverse events, the Investigator is obliged to pursue and provide information as requested by the study monitor/ Sponsor's representative or Sponsor's medical expert in addition to that in the CRF. In general, this will include a description of the adverse event in sufficient detail to allow for a complete medical assessment of the case and independent determination of possible causality. In the case of a subject's death, a summary of available autopsy findings must be submitted

as soon as possible by the Investigator. The Investigator should ensure that information on such cases reported by telephone or other means and information entered in the CRF are accurate and consistent.

Discontinuation of the study due to Adverse Events

The reason for a subject discontinuing from the study will be recorded in the CRF. A discontinuation occurs when an enrolled subject ceases participation in the study, regardless of the circumstances, prior to the completion of the study. A discontinuation must be reported immediately to the sponsor if it is due to a serious adverse event. The final evaluation as required by the Protocol will be performed at the time of study test product discontinuation if medically acceptable or as soon as possible after that. The Investigator will record the reason for study discontinuation and, if possible, provide or arrange for appropriate follow-up and document the cause of the subject's condition.

The association of the adverse events to the study medication can be ascertained by WHO criteria[22] as mentioned below:

**Causality term**	**Assessment criteria***
**Certain**	• Event or laboratory test abnormality, with plausible time relationship to test product intake<br>• Cannot be explained by disease or other drugs<br>• Response to withdrawal plausible (pharmacologically, pathologically)<br>• Event definitive pharmacologically or phenomenologically (i.e. an objective and specific medical disorder or a recognized pharmacological phenomenon)<br>• Rechallenge satisfactory, if necessary
**Probable/ Likely**	• Event or laboratory test abnormality, with reasonable time relationship to test product intake<br>• Unlikely to be attributed to disease or other drugs<br>• Response to withdrawal clinically reasonable<br>• Rechallenge not required
**Possible**	• Event or laboratory test abnormality, with reasonable time relationship to test product intake<br>• Could also be explained by disease or other drugs<br>• Information on test product withdrawal may be lacking or unclear
**Unlikely**	• Event or laboratory test abnormality, with a time to test product intake that makes a relationship improbable (but not impossible)<br>• Disease or other drugs provide plausible explanations

**Conditional/ Unclassified**	• Event or laboratory test abnormality More data for proper assessment needed, or<br>• Additional data under examination
**Unassessable/ Unclassifiable**	• Report suggesting an adverse reaction Cannot be judged because information is insufficient or contradictory Data cannot be supplemented or verified

* All points should be reasonably complied with

The assessment of intensity/severity will be determined based on the investigator's clinical judgment using the following definitions:

Mild: An event that is easily tolerated by the patient, causing minimal discomfort and not interfering with everyday activities.
Moderate: An event that is sufficiently discomforting to interfere with normal everyday activities.
Severe: An event that prevents normal everyday activities.

Note: severity is not the same as 'seriousness', which is defined in SAE above

11 Ethical Considerations

11.1 Good Clinical Practice (GCP)

The procedures set out in this Study Protocol are designed to ensure that M/s Arjuna Natural Extract Limited and Investigator abide by the principles of the Indian GCP, the basis of which lies in the Declaration of Helsinki; in the conduct, evaluation and documentation of this study. Ethics Committee notifications as per Good Clinical Practice Guidelines will also be followed during the conduct of the study.

11.2 Risk / benefit assessment

This study includes interventions offering the prospect of direct health-related benefit to the patient. To establish with certainty that the therapeutic efficacy of Amla Extract (AMX160) in Indian patients is not due to placebo effect; placebo comparator is selected. Patients enrolled in the study will have risk of lack of efficacy of study medication but all the patients will be encouraged for life style modification during the study period.

11.3 Ethics Committee review and communications

This Protocol, corresponding CRF, Informed Consent Documents (including Patient Information Sheet (PIS) and Informed Consent Form (ICF)) will be reviewed by the

registered Institutional Ethics Committee (IEC) / Institutional Review Board (IRB) of each study centre. The first subject must be enrolled only after the IEC/IRB approves the Protocol and other documents for the study. Ethics Committee notifications as per Good Clinical Practice Guidelines will be followed during the conduct of the study.

11.4 Informed consent process

Written informed consent from each of the patient will be taken prior his / her enrolment in the study and will be documented by the Investigator in the source document and will be preserved safely after the completion / termination of the study for at least a period of 5 years if it is not possible to maintain the same permanently The Investigators and / or designated study personnel will explain the IRB / IEC approved Informed Consent Documents (including Patient Information Sheet (PIS) and Informed Consent Form (ICF)) and all relevant details of the study and the study medication to the patient (in English or in a language understandable to the patient) before initiation of the study regarding the purpose, procedures to be carried out, potential hazards, available alternative therapeutic modalities and rights of the patients. They will further be given a description of any foreseeable risks and discomforts. Patient will also be told that they have the right to opt out of the study at any time without having to give reasons if they so wish and without prejudice to further treatment. The patient will be given sufficient time to consider the implications of the study before deciding whether or not to participate in the study. The patient (or LAR in case of minor / mentally challenged) and the principal investigator must sign the informed consent form. A copy of the ICF signed by the investigator and patient and/ or LAR and/ or independent witness will be given to the patient. The patient should have legal capacity and be able to comprehend the nature, meaning, importance and risks of the study and to make up his mind accordingly. If the patient is unable to read and understand the necessary information pertaining to his participation in the study, then, an impartial witness (a person, who is independent of the trial and who cannot be influenced by people involved in the trial) would attend the informed consent process and would explain the contents of the informed consent form, in a language understood by the patient. Date must be mentioned along with the signature of each individual. No patient should be enrolled without obtaining his/ her written informed consent.

11.5 Statement of subject confidentiality (including ownership of data and coding procedures)

The data identifying each study subject by name will be kept confidential and will be accessible only to the Sponsor / Sponsor's authorized representative including QA auditors and / or CRA and if necessary to the IRB / IEC. The subjects will be identified throughout and after the completion of the study by allotted randomization numbers only (as mentioned in subject coding systems in "Test Product Management"

section of the Protocol). The subjects will be informed that their identity will not be revealed except to the above authorities.

11.6 Financial considerations for the subjects

The subjects will not have any financial burden for participating in the study. In the event of an injury occurring to the clinical trial subject, Sponsor will provide free medical management as long as required. In event of a trial related injury, sponsor will provide financial compensation to the subject for the injury. In case of trial related death, his/ her nominee would be entitled for financial compensation.

12 Study Monitoring and Supervision

12.1 Study monitoring

Monitoring and auditing: Standard Operating Procedures developed by Clinical Research Organisation will be followed, in order to comply with Good Clinical Practice guidelines and to ensure acceptability of the study data for registration purposes. Checking of the Case Record Forms (CRFs) for completeness and clarity, and cross-checking with source documents in the presence of the Investigator giving due consideration of data protection and medical confidentiality will be required, and the investigator will assure M/s Arjuna Natural Extract Limited of necessary support at all times. This will be necessary to monitor the progress of the study. Monitoring will be done by the sponsor's designated person. Monitor would visit the study site at least once before, once during and once after the completion of clinical phase of trial at the study site.

12.2 Study materials

M/s Arjuna Natural Extract Limited would supply adequate study medications to the investigator through Clinical Research Organisation. Adequate study materials, including CRFs, study medication supplies will be provided to the Investigator by Clinical Research Organisation. All used and unused study medications and other unused study materials will be collected and must be returned to the sponsor after the clinical phase of the trial has been completed.

12.3 Filling of Case Report Forms

All entries in the Case Record Forms must be made legibly in ballpoint pen (not pencil, felt tip or fountain pen). All data collected during the study must be entered by the Investigator on the CRFs provided. A reasonable explanation must be given by the Investigator for the missing data, if any. The completed CRFs must be signed by the Investigator named in the study Protocol.

If the Investigator authorizes other persons to make entries in the CRF, the names, positions, signatures and initials of those persons must be provided to M/s Arjuna Natural Extract Limited. If a person other than the Investigator enters the findings in the CRF, that person must also sign the CRF.

If corrections are made to entries in the CRF by the Investigator or designate(s), the words or figures must be crossed through, leaving the initial entry legible. The correction must then be dated and initialled. Incorrect entries must not be covered with correcting fluid, obliterated, or made illegible in any way.

CRFs must be kept updated all the times. Arrangements will be made by the sponsor to collect the copy of CRFs on completion. Any missing or erroneous data entry in the CRF should be verified with the investigator and accordingly corrected by the investigator before data entry. Arrangements will be made by the sponsor to collect the copy of those CRFs.

12.4 Data handling & record keeping

All the subjects' findings including demographics will be recorded in source documents. Laboratory reports will also be considered as source documents. All the data captured in these source documents will be entered in the respective CRFs. All raw data and transcribed data forms will be compiled by the study personnel assisting in the study and will be checked, wherever applicable, for completeness. All data related to the study will be in the custody of the Investigator.

12.5 Source data accessibility

The IEC/IRB, the study monitor and QA auditors from Clinical Research Organisation and M/s Arjuna Natural Extract Limited will have the access to the raw data during inspection and audits.

12.6 Data entry and verification

After receiving the copy of CRFs, the data will be checked for inclusion and exclusion criteria and completeness. Major clarifications, if any, will be obtained from the Investigator.

12.7 Archiving

M/s Arjuna Natural Extract Limited will retain a representative sample of the study medication for a minimum period of 5 years after completion of the study. All the data generated during the conduct of study along with site master file and signed informed consent will be archived by the Investigator and a copy of all the documents

will be archived by the sponsor for a minimum period of 5 years after completion of the study.

12.8 Termination of study

The management of M/s Arjuna Natural Extract Limited, Kerala, India reserves the right to discontinue the study at any time. The study may be terminated by the IEC / IRB if there are major violations of ethical considerations or due to any serious adverse event. The reason for discontinuation of the study will be duly documented and the reason will be provided to all the Investigators involved in the study.

13 Test Product Management

13.1 Test Product description and packaging

Study medications consist of Amla Extract (AMX160) 500 mg capsules and matching placebo capsules. The test product would be packed in HDPE bottles each containing 66 capsules Amla Extract (AMX160) 500mg or matching placebo (56 capsules to be used during scheduled visit interval and 10 extra capsules to be used by patients if they fail to come on the scheduled dates). Each patient will be given one container each on visit 2 (Week 0), visit 3 (Week 4) and visit 4 (Week 8).

Composition of Test Products:

Each 500mg capsule contains: 500mg of Amla extract (AMX160)/ Placebo
The test products will be manufactured by M/s Arjuna Natural Extracts Limited.

13.2 The precise dosing regimen

Patients will be instructed to take one Amla Extract (AMX160) 500 mg capsule or matching placebo capsule twice daily after meals as per the random allocation for the entire study duration of 12 weeks.

13.3 Method of packaging, labelling and blinding of Test Product

The study medication would be packed in a total of 3 containers each containing 66 capsules of Amla Extract (AMX160) 500 mg or matching placebo. To maintain blindness of study treatments, placebo capsules resembling in every manner to Amla Extract (AMX160) capsule will be used. The labels of the containers will contain Project No., Randomization No., Name of Test Product, Number of units of the study medication, Batch No, Manufacturing Date, Expiry Date/ Retest date, storage condition, name of manufacturer & sponsor and a statement “For Clinical Trial Use Only”.

13.4 Method of treatment assignment and subject identification coding

The subjects screened for eligibility for enrolment in the trial will be given a screening number. This would be a 10 digit no, first two digits would be site number followed by initials consisting of three digits, starting with 001 for each site. The screening number would be mentioned on the informed consent document of each patient. The patients found eligible for enrolment would be assigned a randomization number, which would consist of a seven digit numerical code, as per the randomization sheet provided to the investigator by the sponsor. Patients who, after consenting to the study, decide not to take part in the study or screen failure patients will retain their screening number, but will not be given randomization number. The next enrolled patient will be given the next randomization number. Each patient enrolled in the study will be given study medication as per the randomization number assigned to him/her. Randomization number given to any patient who discontinues the study or is lost to follow-up will not be allotted to any other subject during the study.

13.5 Storage conditions for Test Product

All study supplies must be stored at room temperature, protected from light and separately from normal hospital / practice stocks in a secure locked facility accessible only to authorized personnel.

13.6 Test Product accountability

The Investigator will confirm receipt of all the study test products in writing. A record of usage of supplies will be kept and the supplies, which are not used in the study, will be returned to the Sponsor after completion of the study. All courier receipts and logs will be maintained at the site for test product accountability. The study medications will be supplied to study patients under the responsibility of the investigator and the record of same must be maintained. All supplies must be accounted for at the end of the study period.

13.7 Procedures for handling unused Test Products

Unused study test product supplies will be returned to M/s Arjuna Natural Extract Limited after completion of the study. Any discrepancy between the amounts dispensed and returned must be explained by investigator in the CRF.

14 Data Analysis

14.1 Sample size calculation

The sample size of 98 patients was calculated based on the following assumptions. Considering a mean reduction in TG between the group as 51 [29], the standard deviation as 76 [29] (effect Size 0.67 rounded to 0.7) with 5% alpha level (two sided) and power of 90%, at least 44 patients would be needed in each group. Assuming the dropout rate of 10% a total of 49 patients were enrolled in the study in each group. The enrolled patients would be assigned to either of the 2 treatment groups according to a centralized computer generated randomization plan in 1:1 ratio.

Data Entry and Data Validation

After checking and coding the completed CRFs, the data entry will be undertaken. A single time data entry with manual checking of the data will be done.

14.2 Missing, Unused or Spurious Data

Clarifications, wherever possible, will be obtained from the respective Investigator for any missing data or for any illegible entry. Unused or spurious data will be kept in due confidentiality for a period of 5 years along with the other documents. Missing data shall not be imputed.

14.3 Statistical analysis

14.4.1 Immunogenicity Population

Per Protocol Population

All the randomized patients who will complete the trial as per the defined protocol (including deviations) with both baseline and end of the study laboratory assessments will be considered in the Per Protocol analysis.

Modified Intention to Treat Population (mITT)

All the randomized patients with baseline laboratory investigations and those who have completed at least one post randomization visit i.e visit 3 (week 4) with laboratory evaluation including patients with protocol violations will be considered in the mITT analysis.

14.4.2 Safety Population

Safety population will include all the randomized patients who have been dispensed study medication and have completed at least one post randomization visit i.e. visit 3 (week 4) will be considered for safety evaluation.

14.4.3 Data Analysis

The data collected from the CRFs will be analysed for demographics, efficacy & safety. Data will be presented as mean ± SD/SE or number (percentage). Descriptive statistics will be used for different variables at baseline. P value of < 0.05 will be considered as statistically significant. Standard statistical tests will be used to analyse the data obtained. For efficacy analysis both, modified Intention To Treat (mITT) analysis and Per Protocol (PP) analysis will be done. The PP analysis will be considered as definitive, while the mITT analysis will be considered as supportive during the trial analysis.

15 Financial Considerations

The conduct of the study will not impose any financial burden on the Investigator(s) and the trial centre(s). The sponsor, M/s Arjuna Natural Extracts Limited, declares to bear all the expenses pertaining to the conduct of the study.

16 Publication Policy

The data generated during the study is sponsor's property. In this multicentre study, the sponsor reserves the right to publish the results of the trial in a scientific journal on behalf of all the Investigators and due acknowledgement would be made to all the Investigators participating in the study.

17 Sponsor's Representative / Medical Expert

Name: Dr Binu.T.Kuruvilla
Degrees: M.B.B.S
Title: AGM R&D
Address: M/s Arjuna Natural Extracts Limited

18 Appendices

18.1 World Medical Association - Declaration of Helsinki

18.2 Randomization Schedule

18.3 Serious Adverse Event (SAE) Reporting Form

18.4 Unscheduled Visit Form

18.4 Overall Tolerability Form

DECLARATION OF HELSINKI

Policy

WORLD MEDICAL ASSOCIATION DECLARATION OF HELSINKI Ethical Principles for Medical Research Involving Human Subjects
Adopted by the 18th WMA General Assembly, Helsinki, Finland, June 1964, and amended by the 29th WMA General Assembly, Tokyo, Japan, October 1975
35th WMA General Assembly, Venice, Italy, October 1983 41st WMA General Assembly, Hong Kong, September 1989
48th WMA General Assembly, Somerset West, Republic of South Africa, October 1996 and the
52nd WMA General Assembly, Edinburgh, Scotland, October 2000 Note of Clarification on Paragraph 29 added by the WMA General Assembly, Washington 2002
Note of Clarification on Paragraph 30 added by the WMA General Assembly, Tokyo 2004.

A. INTRODUCTION

1. The World Medical Association has developed the Declaration of Helsinki as a statement of ethical principles to provide guidance to physicians and other participants in medical research involving human subjects. Medical research involving human subjects includes research on identifiable human material or identifiable data.

2. It is the duty of the physician to promote and safeguard the health of the people. The physician's knowledge and conscience are dedicated to the fulfillment of this duty.

3. The Declaration of Geneva of the World Medical Association binds the physician with the words, "The health of my patient will be my first consideration," and the International Code of Medical Ethics declares that, "A physician shall act only in the patient's interest when providing medical care which might have the effect of weakening the physical and mental condition of the patient."

4. Medical progress is based on research which ultimately must rest in part on experimentation involving human subjects.

5. In medical research on human subjects, considerations related to the well-being of the human subject should take precedence over the interests of science and society.

6. The primary purpose of medical research involving human subjects is to improve prophylactic, diagnostic and therapeutic procedures and the understanding of the etiology and pathogenesis of disease. Even the best proven prophylactic, diagnostic, and therapeutic methods must continuously be challenged through research for their effectiveness, efficiency, accessibility and quality.

7. In current medical practice and in medical research, most prophylactic, diagnostic and therapeutic procedures involve risks and burdens

8. Medical research is subject to ethical standards that promote respect for all human beings and protect their health and rights. Some research populations are vulnerable and need special protection. The particular needs of the economically and medically disadvantaged must be recognized. Special attention is also required for those who cannot give or refuse consent for themselves, for those who may be subject to giving consent under duress, for those who will not benefit personally from the research and for those for whom the research is combined with care.

9. Research Investigators should be aware of the ethical, legal and regulatory requirements for research on human subjects in their own countries as well as applicable international requirements. No national ethical, legal or regulatory requirement should be allowed to reduce or eliminate any of the protections for human subjects set forth in this Declaration.

B. BASIC PRINCIPLES FOR ALL MEDICAL RESEARCH

10. It is the duty of the physician in medical research to protect the life, health, privacy, and dignity of the human subject.

11. Medical research involving human subjects must conform to generally accepted scientific principles, be based on a thorough knowledge of the scientific literature, other relevant sources of information, and on adequate laboratory and, where appropriate, animal experimentation.

12. Appropriate caution must be exercised in the conduct of research which may affect the environment, and the welfare of animals used for research must be respected.

13. The design and performance of each experimental procedure involving human subjects should be clearly formulated in an experimental protocol. This protocol should be submitted for consideration, comment, guidance, and where appropriate, approval to a specially appointed ethical review committee, which must be independent of the investigator, the sponsor or any other kind of undue influence. This independent committee should be in conformity with the laws and regulations of the country in which the research experiment is performed. The committee has the right to monitor ongoing trials. The researcher has the obligation to provide monitoring information to the committee, especially any serious adverse events. The researcher should also submit to the committee, for review, information regarding funding, sponsors, institutional affiliations, other potential conflicts of interest and incentives for subjects.

14. The research protocol should always contain a statement of the ethical considerations involved and should indicate that there is compliance with the the principles enunciated in this Declaration.

15. Medical research involving human subjects should be conducted only by scientifically qualified persons and under the supervision of a clinically competent medical person. The responsibility for the human subject must always rest with a medically qualified person and never rest on the subject of the research, even though the subject has given consent.

16. Every medical research project involving human subjects should be preceded by careful assessment of predictable risks and burdens in comparison with foreseeable benefits to the subject or to others. This does not preclude the participation of healthy volunteers in medical research. The design of all studies should be publicly available

17. Physicians should abstain from engaging in research projects involving human subjects unless they are confident that the risks involved have been adequately assessed and can be satisfactorily managed. Physicians should cease any investigation if the risks are found to outweigh the potential benefits or if there is conclusive proof of positive and beneficial results.

18. Medical research involving human subjects should only be conducted if the importance of the objective outweighs the inherent risks and burdens to the subject. This is especially important when the human subjects are healthy volunteers.

19. Medical research is only justified if there is a reasonable likelihood that the populations in which the research is carried out stand to benefit from the results of the research.

20. The subjects must be volunteers and informed participants in the research project.

21. The right of research subjects to safeguard their integrity must always be respected. Every precaution should be taken to respect the privacy of the subject, the confidentiality of the patient's information and to minimize the impact of the study on the subject's physical and mental integrity and on the personality of the subject.

22. In any research on human beings, each potential subject must be adequately informed of the aims, methods, sources of funding, any possible conflicts of interest, institutional affiliations of the researcher, the anticipated benefits and potential risks of the study and the discomfort it may entail. The subject should be informed of the right to abstain from participation in the study or to withdraw consent to participate at any time without reprisal. After ensuring that the subject has understood the information, the physician should then obtain the subject's freely-given informed consent, preferably in writing. If the consent cannot be obtained in writing, the nonwritten consent must be formally documented and witnessed

23. When obtaining informed consent for the research project the physician should be particularly cautious if the subject is in a dependent relationship with the physician or may consent under duress. In that case the informed consent should be obtained by a well informed physician who is not engaged in the investigation and who is completely independent of this relationship.

24. For a research subject who is legally incompetent, physically or mentally incapable of giving consent or is a legally incompetent minor, the investigator must obtain informed consent from the legally authorized representative in accordance with applicable law. These groups should not be included in research unless the research is necessary to promote the health of the population represented and this research cannot instead be performed on legally competent persons.

25. When a subject deemed legally incompetent, such as a minor child, is able to give assent to decisions about participation in research, the investigator must obtain that assent in addition to the consent of the legally authorized representative.

26. Research on individuals from whom it is not possible to obtain consent, including proxy or advance consent, should be done only if the physical/mental condition that prevents obtaining informed consent is a necessary characteristic of the research population. The specific reasons for involving research subjects with a condition that renders them unable to give informed consent should be stated in the experimental protocol for consideration and and approval of the obtained as soon as possible from the individual or a legally authorized surrogate.

27. Both authors and publishers have ethical obligations. In publication of the results of research, the investigators are obliged to preserve the accuracy of the results. Negative as well as positive results should be published or otherwise publicly available. Sources of funding, institutional affiliations and any possible conflicts of interest should be declared in the publication. Reports of experimentation not in accordance with the principles laid down in this Declaration should not be accepted for publication.

C. ADDITIONAL PRINCIPLES FOR MEDICAL RESEARCH COMBINED WITH MEDICAL CARE

28. The physician may combine medical research with medical care, only to the extent that the research is justified by its potential prophylactic, diagnostic or therapeutic value. When medical research is combined with medical care, additional standards apply to protect the subjects who are research subjects.

29. The benefits, risks, burdens and effectiveness of a new method should be tested against those of the best current prophylactic, diagnostic, and therapeutic methods. This does not exclude the use of placebo, or no treatment, in studies where no proven prophylactic, diagnostic or therapeutic method exists.

30. At the conclusion of the study, every patient entered into the study should be assured of access to the best proven prophylactic, diagnostic and therapeutic methods identified by the study.

31. The physician should fully inform the patient which aspects of the care are related to the research. The refusal of a patient to participate in a study must never interfere with the patient physician relationship.

In the treatment of a patient, where proven prophylactic, diagnostic and therapeutic methods do not exist or have been ineffective, the physician, with informed consent from the patient, must be free to use unproven or new prophylactic, diagnostic and therapeutic measures, if in the physician's judgment it offers hope of saving life, re-establishing health or alleviating suffering. Where possible, these measures should be made the object of research, designed to evaluate their safety and efficacy. In all cases, new information should be recorded and, where appropriate, published. The other relevant guidelines of this Declaration should be followed.

1 Note of clarification on paragraph 29 of the WMA Declaration of Helsinki

The WMA hereby reaffirms its position that extreme care must be taken in making use of a placebo-controlled trial and that in general this methodology should only be used in the absence of existing proven therapy. However, a placebo-controlled trial may be ethically acceptable, even if proven therapy is available, under the following circumstances: ----Where for compelling and scientifically sound methodological reasons its use is necessary to determine the efficacy or safety of a prophylactic, diagnostic or therapeutic method; or

- Where a prophylactic, diagnostic or therapeutic method is being investigated for a minor condition and the subjects who receive placebo will not be subject to any additional risk of or irreversible harm. All other provisions of the Declaration of Helsinki must be adhered to, especially the need for appropriate ethical and scientific review.

2 Note of clarification on paragraph 30 of the WMA Declaration of Helsinki

The WMA hereby reaffirms its position that it is necessary during the study planning process to identify post-trial access by study participants to prophylactic, diagnostic and therapeutic procedures identified as beneficial in the study or access to other appropriate care. Post-trial access arrangements or other care must be described in the study protocol so the ethical review committee may consider such arrangements during its review**.**

Randomization Schedule

SUBJECT		
**Subject No**	**Randomization Code**	**Sex**
**01**		
**02**		
**03**		
**04**		
**05**		
**06**		
**07**		
**08**		
**09**		
**10**		
**11**		
**12**		
**13**		
**14**		
**15**		
**16**		
**17**		
**18**		
**19**		
**20**		
**21**		
**22**		
**23**		
**24**		
**25**		
**26**		
**27**		
**28**		
**29**		
**30**		
**31**		
**32**		
**33**		
**34**		
**35**		
**36**		
**37**		
**38**		
**39**		
**40**		
**41**		
**42**		
**43**		
**44**		
**45**		
**46**		
**47**		
**48**		
**49**		
**50**		
**51**		
**52**		
**53**		
**54**		
**55**		

**56**		
**57**		
**58**		
**59**		
**60**		
**61**		
**62**		
**63**		
**64**		
**65**		
**66**		
**67**		
**68**		
**69**		
**70**		
**71**		
**72**		
**73**		
**74**		
**75**		
**76**		
**77**		
**78**		
**80**		
**81**		
**82**		
**83**		
**84**		
**85**		
**86**		
**87**		
**88**		
**89**		
**90**		
**91**		
**92**		
**93**		
**94**		
**95**		
**96**		
**97**		
**98**		

SERIOUS ADVERSE EVENTS (SAE) LOG

Date of Submission to Sponsor: ____________________

Date of Submission to EC: ____________________

SAE report:

- SAE report #:____

Type of report: ☐ **Initial** ☐ **Follow up**

SAE Information:

- Type of SAE:____
- Severity: ____

Start Date: ________ _________ _________
DD MMM YYYY
SAE onset time: ____________
H H

- Place of Occurrence:____
- Was this SAE unexpected? ☐Yes ☐No
- Diagnosis:____
- Brief Description:____

____

____

- Suspected Drug: ________________________ (**Mention Generic Name**)
- Indication(s) for the suspect drug was prescribed or tested: __________________
- Dosage form & strength: ____

Route of Administration: ____

Start Date and Time of Treatment: ________ ________ ________
DD MMM YYYY

Stop Date and Time of Treatment: ________ ________ ________
DD MMM YYYY

Outcome of SAE:

☐ Disability/ Incapability:

☐ Life-threatening:

☐ Congenital anomaly/birth defect:

☐ Hospitalization-initial or prolonged:

Admission Date: ________ ________ ________
DD MMM YYYY

Discharge Date: ________ ________ ________
DD MMM YYYY

☐ Required intervention to prevent permanent impairment

☐ Other (Please explain):

____

UNSCHEDULED VISIT

Reason/s for Unscheduled Visit:

1. Due to missed Visit ☐
2. Due to Adverse Events ☐
   Record details in Adverse Events Log
3. Due to concurrent clinical condition ☐
4. Any other ☐
   Specify below

____

____

Investigator Comments:

____

____

OVERALL TOLERABILITY EVALUATION

*	Grade	Description
☐	Excellent	No adverse event
☐	Good	Mild adverse event(s) reported which subsided with or without medication and did not necessitate stoppage of study medication
☐	Fair	Moderate adverse event(s) reported which subsided with or without medication and did not necessitate stoppage of study medication
☐	Poor	Severe or serious adverse event(s) which necessitated stoppage of study medication

Check as appropriate

19. Bibliography

1. Assman G, Schulte H. Relation of high density cholesterol and triglycerides to incidence of atherosclerotic coronary artery disease (The PROCAM experience). Am J Cardiol 1992;70:733-7

2. Mukherjee S. Therapeutic management of hyperlipidemia - Rationale and outcome. Assoc Physicians India 2002:315-30

3. Anila L, Vijayalakshmi NR. Flavonoids from Emblica officinalis and Mangifera indica-effectiveness for dyslipidemia. J Ethnopharmacol 2002; 79:81-7.

4. Antony B, Merina B, Sheeba V, Mukkadan J. Effect of standardized Amla extract on atherosclerosis and dyslipidemia. Ind J Pharm Sci 2006; 68 (4): 437-41.

5. Mishra M, Pathak UN, Khan AB. Emblica officinalis Gaertn and serum cholesterol level in experimental rabbits. Br J Exp Pathol 1981; 62 (5): 526-8.

6. Mathur R, Sharma A, Dixit VP, Varma M. Hypolipidaemic effect of fruit juice of Emblica officinalis in cholesterol-fed rabbits. J Ethnopharmacol 1996; 50(2): 61-8.

7. Thakur CP, Mandal K. Effect of Emblica officinalis on cholesterol-induced atherosclerosis in rabbits. Ind J Med Res 1984; 79: 142-6.

8. Annu J, Pandey M, Kapoor S, Saroja R. Effect of the Indian gooseberry (amla) on serum cholesterol levels in men aged 35-55 years. Eur J Clin Nutr 1988; 42: 939-44.

9. Antony B, Benny M, Kaimal TNB. A Pilot clinical study to evaluate the effect of Emblica officinalis extract (Amlamax™) on markers of systemic inflammation and dyslipidaemia. Ind J Clin Biochemistry 2008; 23(4):378-381

10. Antony B, Merina B, Sheeba V. Amlamax in the management of dyslipidemia in humans. Indian J Pharm Sci. 2008 Jul-Aug;70(4):504-7

11. Gopa B, Bhatt J, Hemavathi KG. A comparative clinical study of hypolipidemic efficacy of *Amla* (*Emblica officinalis*) with 3-hydroxy-3-methylglutaryl-coenzyme-A reductase inhibitor simvastatin. Indian J Pharmacol 2012;44:238-42

12. M. Dobiasova. Atherogenic index of plasma [log(triglycerides/HDL-cholesterol)]: theoretical and practical implications. Clin Chem, 50 (2004), pp. 1113–1115

13. Kannan G, Rajendran NN, Murthy JSN, Rani V. The Predictability of Apo B, Apo A1, ApoB/ApoA1 as better Markers for CAD in South Indian Population Int J Science and Research. 2014;3(6):240-249

14. Nygård O, Nordrehaug JE, Refsum H, Ueland PM, Farstad M, Vollset SE. Plasma homocysteine levels and mortality in patients with coronary artery disease. N Engl J Med. 1997 Jul 24;337(4):230-6.

15. Lee BJ, Tseng YF, Yen CH, Lin PT Effects of coenzyme Q10 supplementation (300 mg/day) on antioxidation and anti-inflammation in coronary artery disease patients during statins therapy: a randomized, placebo-controlled trial. Nutr J. 2013 Nov 6;12(1):142

16. Auer J, Berent R, Lassnig E, Eber B. C-reactive protein and coronary artery disease. Jpn Heart J. 2002 Nov;43(6):607-19.

17. Akhtar MS, Ramzan A, Ali A, Ahmad M. Effect of Amla fruit (Emblica officinalis Gaertn.) on blood glucose and lipid profile of normal subjects and type 2 diabetic patients. Int J Food Sci Nutr. 2011 Sep;62(6):609-16.

18. U.S. Department Of Health And Human Services. ATP III Guidelines At-A-Glance Quick Desk Reference. May 2001. NIH Publication No. 01-3305

19. Honda A, Mizokami Y, Matsuzaki Y, Ikegami T, Doy M, Miyazaki H. Highly sensitive assay of HMG-CoA reductase activity by LC-ESI-MS/MS. J Lipid Res. 2007 May;48(5):1212-20.
20. British Cardiovascular Society. Recording a standard 12-lead electrocardiogram – An approved methodology. The society for Cardiovascular Science and Technology. 2013 February. Available from http://www.scst.org.uk/resources/consensus_guideline_for_recording_a_12_lead_ecg_Rev_072010b.pdf
21. McGuffin M, Hobbs C, Upton R, Goldberg A, editors. 1997. American Herbal Products Association's Botanical Safety Handbook. Boca Raton (FL): CRC Press
22. The use of the WHO–UMC system for standardised case causality assessment. Accessed from: http://who-umc.org/Graphics/24734.pdf [last accessed on 2014 May 09]
23. Joseph S, Santhosh D, Udupa AL, Gupta S, Ojeh N, Rathnakar UP, et al Hypolipidemic Activity of Phyllanthus Emblica Linn (Amla) & Trigonella Foenum Graecum (Fenugreek) Combination In Hypercholesterolemic Subjects – A Prospective, Randomised, Parallel, Open-Label, Positive Controlled Study. Asian Journal of Biochemical and Pharmaceutical Research 2012;1(*2): 225-230*
