# Supplementary material for: A randomized, double blind, placebo controlled, multicenter clinical trial to assess the efficacy and safety of Emblica officinalis extract in patients with dyslipidemia
Source: BMC Complement Altern Med. 2019 Jan 22;19:27. doi: 10.1186/s12906-019-2430-y (PMC6341673; doi:10.1186/s12906-019-2430-y)
Supplement: Supplementary file 5 — Full data of all visits. (DOCX 25 kb) [file 12906_2019_2430_MOESM5_ESM.docx]

**Full data of all visits**

**Table: Efficacy parameters**

|  | | **Treatment** | |
| --- | --- | --- | --- |
| **Parameter /Statistics** | **Visit** | **Amla extract** | **Placebo** |
| **Total cholesterol mg/dL** | | | |
| N | Visit_1 | 49 | 49 |
| Mean(SD) | Visit_1 | 231.7 (27.03) | 225.7 (29.03) |
| Median | Visit_1 | 223.0 | 216.0 |
| N | Visit_3 | 49 | 49 |
| Mean(SD) | Visit_3 | 208.0 (44.36) | 215.7 (36.50) |
| Median | Visit_3 | 197.0 | 210.0 |
| N | Visit_4 | 49 | 49 |
| Mean(SD) | Visit_4 | 198.0 (62.01) | 211.0 (32.36) |
| Median | Visit_4 | 174.0 | 210.0 |
| N | Visit_5 | 49 | 49 |
| Mean(SD) | Visit_5 | 177.0 (60.04) | 212.6 (31.78) |
| Median | Visit_5 | 151.0 | 210.0 |
| **Triglycerides mg/dL** | | | |
| N | Visit_1 | 49 | 49 |
| Mean(SD) | Visit_1 | 261.1 (74.13) | 247.6 (57.70) |
| Median | Visit_1 | 223.2 | 225.0 |
| N | Visit_3 | 49 | 49 |
| Mean(SD) | Visit_3 | 217.9 (136.7) | 234.1 (92.91) |
| Median | Visit_3 | 180.0 | 220.0 |
| N | Visit_4 | 49 | 49 |
| Mean(SD) | Visit_4 | 188.0 (81.15) | 227.9 (100.3) |
| Median | Visit_4 | 166.0 | 216.0 |
| N | Visit_5 | 49 | 49 |
| Mean(SD) | Visit_5 | 171.9 (86.51) | 210.5 (65.27) |
| Median | Visit_5 | 142.0 | 201.0 |
| **High Density Lipoproteins mg/dL** | | | |
| N | Visit_1 | 49 | 49 |
| Mean(SD) | Visit_1 | 43.7 ( 7.45) | 43.4 ( 7.34) |
| Median | Visit_1 | 44.0 | 41.2 |
| N | Visit_3 | 49 | 49 |
| Mean(SD) | Visit_3 | 42.3 ( 7.99) | 41.3 ( 6.84) |
| Median | Visit_3 | 40.0 | 41.0 |
| N | Visit_4 | 49 | 49 |
| Mean(SD) | Visit_4 | 43.3 ( 9.95) | 43.1 (7.01) |
| Median | Visit_4 | 41.0 | 41.2 |
| N | Visit_5 | 49 | 49 |
| Mean(SD) | Visit_5 | 39.3 ( 8.50) | 41.7 (6.09) |
| Median | Visit_5 | 37.0 | 41.0 |
| **Low Density Lipoproteins mg/dL** | | | |
| N | Visit_1 | 49 | 49 |
| Mean(SD) | Visit_1 | 140.0 (19.66) | 132.2 (20.82) |
| Median | Visit_1 | 136.0 | 126.0 |
| N | Visit_3 | 49 | 49 |
| Mean(SD) | Visit_3 | 128.2 (34.61) | 125.8 (20.37) |
| Median | Visit_3 | 117.0 | 124.0 |
| N | Visit_4 | 49 | 49 |
| Mean(SD) | Visit_4 | 122.2 (45.85) | 125.1 (25.73) |
| Median | Visit_4 | 112.0 | 121.0 |
| N | Visit_5 | 49 | 49 |
| Mean(SD) | Visit_5 | 111.5 (43.22) | 126.1 (27.02) |
| Median | Visit_5 | 97.0 | 121.0 |
| **Very Low Density Lipoproteins mg/dL** | | | |
| N | Visit_1 | 49 | 49 |
| Mean(SD) | Visit_1 | 51.7 (15.9) | 49.6 (11.68) |
| Median | Visit_1 | 44.0 | 45 |
| N | Visit_3 | 48 | 49 |
| Mean(SD) | Visit_3 | 40.5 (15.43) | 46.8 (18.87) |
| Median | Visit_3 | 36 | 44 |
| N | Visit_4 | 49 | 49 |
| Mean(SD) | Visit_4 | 38(16.3) | 44.84(20.2) |
| Median | Visit_4 | 34.0 | 42.2 |
| N | Visit_5 | 49 | 49 |
| Mean(SD) | Visit_5 | 34.0 (17.32) | 41.8 (12.89) |
| Median | Visit_5 | 28 | 40.0 |
| **Fasting Plasma Glucose mg/dL** | | | |
| N | Visit_1 | 49 | 49 |
| Mean(SD) | Visit_1 | 90.6 (26.83) | 91.9 (22.12) |
| Median | Visit_1 | 84.0 | 87.0 |
| N | Visit_3 | 49 | 49 |
| Mean(SD) | Visit_3 | 93.3 (21.32) | 101.5 (29.43) |
| Median | Visit_3 | 87.0 | 92.0 |
| N | Visit_4 | 49 | 49 |
| Mean(SD) | Visit_4 | 93.9 (19.39) | 97.1 (21.12) |
| Median | Visit_4 | 90.0 | 96.0 |
| N | Visit_5 | 49 | 49 |
| Mean(SD) | Visit_5 | 94.4 (19.41) | 100.4 (22.97) |
| Median | Visit_5 | 90.0 | 96.0 |
| **Atherogenic index of Plasma** | | | |
| N | Visit_1 | 49 | 49 |
| Mean(SD) | Visit_1 | 0.43(0.14) | 0.41(0.12) |
| Median | Visit_1 | 0.38 | 0.38 |
| N | Visit_3 | 49 | 49 |
| Mean(SD) | Visit_3 | 0.33(0.21) | 0.40(0.15) |
| Median | Visit_3 | 0.32 | 0.39 |
| N | Visit_4 | 49 | 49 |
| Mean(SD) | Visit_4 | 0.27(0.20) | 0.36(0.18) |
| Median | Visit_4 | 0.27 | 0.36 |
| N | Visit_5 | 49 | 49 |
| Mean(SD) | Visit_5 | 0.26(0.20) | 0.35(0.15) |
| Median | Visit_5 | 0.25 | 0.36 |

|  | | **Treatment** | |
| --- | --- | --- | --- |
| **Parameter /Statistics** | **Visit** | **Amla extract** | **Placebo** |
| **Apo lipoprotein A1 mg/dl** | | | |
| N | Visit_2 | 49 | 49 |
| Mean(SD) | Visit_2 | 1.15 ( 0.17) | 1.24 ( 0.22) |
| Median | Visit_2 | 1.14 | 1.2 |
| Min, Max | Visit_2 | 0.7, 1.51 | 0.96, 2 |
| N | Visit_5 | 49 | 48 |
| Mean(SD) | Visit_5 | 1.19( 0.16) | 1.21 (0.12) |
| Median | Visit_5 | 1.2 | 1.2 |
| Min, Max | Visit_5 | 0.81, 1.6 | 1, 1.5 |
| **Apo lipoprotein B mg/dl** | | | |
| N | Visit_2 | 49 | 49 |
| Mean(SD) | Visit_2 | 1.13 ( 0.27) | 1.05 (0.21) |
| Median | Visit_2 | 1.08 | 1.04 |
| Min, Max | Visit_2 | 0.7, 2 | 0.68, 1.58 |
| N | Visit_5 | 49 | 48 |
| Mean(SD) | Visit_5 | 1.12 ( 0.30) | 1.0 3( 0.25) |
| Median | Visit_5 | 1.06 | 1.03 |
| Min, Max | Visit_5 | 0.5, 2 | 0.44, 1.58 |
| **Ratio of Apo lipoprotein B to Apo lipoprotein A1** | | | |
| N | Visit_2 | 49 | 49 |
| Mean(SD) | Visit_2 | 1.0 ( 0.34) | 0.87 ( 0.22) |
| Median | Visit_2 | 0.94 | 0.87 |
| Min, Max | Visit_2 | 0.625, 2.9 | 0.32, 1.4 |
| N | Visit_5 | 49 | 48 |
| Mean(SD) | Visit_5 | 0.95 ( 0.29) | 0.85 ( 0.20) |
| Median | Visit_5 | 0.88 | 0.83 |
| Min, Max | Visit_5 | 0.5, 2.4 | 0.32, 1.3 |
| **hs-CRP mg/L** | | | |
| N | Visit_2 | 49 | 49 |
| Mean(SD) | Visit_2 | 3.83 ( 5.81) | 4.48( 5.99) |
| Median | Visit_2 | 1.91 | 2.58 |
| Min, Max | Visit_2 | 0.25, 38 | 0.24, 37 |
| N | Visit_5 | 49 | 48 |
| Mean(SD) | Visit_5 | 3.88 ( 6.23) | 6.09 ( 7.63) |
| Median | Visit_5 | 1.75 | 4.63 |
| Min, Max | Visit_5 | 0.21, 39 | 0.16, 39 |
| **Homocystiene** μmol/L | | | |
| N | Visit_2 | 49 | 49 |
| Mean(SD) | Visit_2 | 23.58 (14.57) | 21.4 (11.10) |
| Median | Visit_2 | 19.44 | 18.2 |
| Min, Max | Visit_2 | 9.18, 82 | 6.79, 67 |
| N | Visit_5 | 49 | 48 |
| Mean(SD) | Visit_5 | 19.17 ( 8.93) | 17.18 ( 5.8) |
| Median | Visit_5 | 16.54 | 16.82 |
| Min, Max | Visit_5 | 8.82, 57 | 5.22, 29.5 |
| **Glycosylated Haemoglobin %** | | | |
| N | Visit_2 | 49 | 49 |
| Mean(SD) | Visit_2 | 6.27 ( 1.04) | 6.22 ( 1.12) |
| Median | Visit_2 | 6.0 | 5.9 |
| Min, Max | Visit_2 | 5, 11 | 5, 10 |
| N | Visit_5 | 49 | 48 |
| Mean(SD) | Visit_5 | 6.46 ( 1.72) | 6.21 ( 1.17) |
| Median | Visit_5 | 5.8 | 5.9 |
| Min, Max | Visit_5 | 5, 11 | 4.5, 10 |
| **TSH μIU/ml** | | | |
| N | Visit_2 | 49 | 49 |
| Mean(SD) | Visit_2 | 2.43 ( 1.50) | 2.53 ( 2.58) |
| Median | Visit_2 | 2.07 | 2.16 |
| Min, Max | Visit_2 | 0.01, 8 | 0.25, 17 |
| N | Visit_5 | 48 | 48 |
| Mean(SD) | Visit_5 | 3.19 ( 4.45) | 3.72 ( 5.67) |
| Median | Visit_5 | 2.29 | 2.39 |
| Min, Max | Visit_5 | 0.38, 29 | 0.73, 34 |
