# Supplementary material for: A randomized, double blind, placebo controlled, multicenter clinical trial to assess the efficacy and safety of Emblica officinalis extract in patients with dyslipidemia
Source: BMC Complement Altern Med. 2019 Jan 22;19:27. doi: 10.1186/s12906-019-2430-y (PMC6341673; doi:10.1186/s12906-019-2430-y)
Supplement: Supplementary file 6 — Safety parameters. (DOCX 28 kb) [file 12906_2019_2430_MOESM6_ESM.docx]

**Safety parameters**

**Table: Hematology data**

|  | | Treatment | |
| --- | --- | --- | --- |
| Parameter /Statistics | Visit | Amla extract | Placebo |
| **Haemoglobin-mg/dl** | | | |
| N | Visit_1 | 49 | 49 |
| Mean(SD) | Visit_1 | 14.0 ( 2.34) | 13.9 ( 2.04) |
| Median | Visit_1 | 14.1 | 13.8 |
| Min, Max | Visit_1 | 3, 17 | 8, 18 |
| N | Visit_5 | 47 | 47 |
| Mean(SD) | Visit_5 | 15.1 ( 3.75) | 13.3 ( 2.97) |
| Median | Visit_5 | 14.4 | 13.2 |
| Min, Max | Visit_5 | 7, 26 | 7, 21 |
| **Total Leukocyte Count-x10^3^/μL** | | | |
| N | Visit_1 | 49 | 49 |
| Mean(SD) | Visit_1 | 7.9 ( 2.57) | 8.5 ( 2.81) |
| Median | Visit_1 | 7.8 | 7.8 |
| Min, Max | Visit_1 | 3, 16 | 3, 19 |
| N | Visit_5 | 45 | 43 |
| Mean(SD) | Visit_5 | 8.6 ( 2.22) | 8.5 ( 2.96) |
| Median | Visit_5 | 8.2 | 7.9 |
| Min, Max | Visit_5 | 5, 15 | 2, 17 |
| **Absolute Neutrophil Count (ANC)%** | | | |
| N | Visit_1 | 49 | 49 |
| Mean(SD) | Visit_1 | 52.0 (12.62) | 53.8 (10.49) |
| Median | Visit_1 | 53.0 | 54.0 |
| Min, Max | Visit_1 | 24, 76 | 23, 81 |
| N | Visit_5 | 45 | 43 |
| Mean(SD) | Visit_5 | 55.1 ( 7.45) | 55.0 (11.68) |
| Median | Visit_5 | 56.0 | 55.0 |
| Min, Max | Visit_5 | 40, 73 | 2, 81 |
| Platelet Count |  |  |  |
| N | Visit_1 | 49 | 49 |
| Mean(SD) | Visit_1 | 293.2 (63.99) | 305.4 (75.24) |
| Median | Visit_1 | 301.0 | 310.0 |
| Min, Max | Visit_1 | 156, 414 | 148, 478 |
| N | Visit_5 | 47 | 47 |
| Mean(SD) | Visit_5 | 291.6 (80.36) | 281.1 (118.0) |
| Median | Visit_5 | 282.0 | 287.0 |
| Min, Max | Visit_5 | 161, 450 | 48, 621 |

**Table: Biochemistry parameters**

|  | | Treatment | |
| --- | --- | --- | --- |
| Parameter /Statistics | Visit | Amla extract | Placebo |
| **Blood Urea mg/dl** | | | |
| N | Visit_1 | 49 | 49 |
| Mean(SD) | Visit_1 | 9.7 ( 3.42) | 10.2 ( 5.39) |
| Median | Visit_1 | 9.0 | 9.0 |
| Min, Max | Visit_1 | 4, 20 | 5, 40 |
| N | Visit_5 | 49 | 48 |
| Mean(SD) | Visit_5 | 10.7 ( 4.22) | 10.2 ( 3.58) |
| Median | Visit_5 | 11.0 | 10.0 |
| Min, Max | Visit_5 | 1, 24 | 5, 20 |
| **Serum Creatinine-mg/dl** | | | |
| N | Visit_1 | 49 | 49 |
| Mean(SD) | Visit_1 | 0.8 ( 0.15) | 0.9 ( 0.60) |
| Median | Visit_1 | 0.8 | 0.8 |
| Min, Max | Visit_1 | 1, 1 | 1, 5 |
| N | Visit_5 | 49 | 48 |
| Mean(SD) | Visit_5 | 0.8 ( 0.14) | 0.9 ( 0.18) |
| Median | Visit_5 | 0.8 | 0.8 |
| Min, Max | Visit_5 | 1, 1 | 1, 2 |
| **Serum Bilirubin-mg/dl** | | | |
| N | Visit_1 | 49 | 49 |
| Mean(SD) | Visit_1 | 0.6 ( 0.32) | 0.6 ( 0.28) |
| Median | Visit_1 | 0.4 | 0.5 |
| Min, Max | Visit_1 | 0, 2 | 0, 2 |
| N | Visit_5 | 49 | 48 |
| Mean(SD) | Visit_5 | 0.7 ( 0.37) | 0.7 ( 0.40) |
| Median | Visit_5 | 0.6 | 0.5 |
| Min, Max | Visit_5 | 0, 2 | 0, 2 |
| **SGOT U/L** | | | |
| N | Visit_1 | 49 | 49 |
| Mean(SD) | Visit_1 | 27.2 (10.23) | 27.4 ( 9.35) |
| Median | Visit_1 | 25.0 | 25.0 |
| Min, Max | Visit_1 | 14, 73 | 16, 57 |
| N | Visit_5 | 49 | 48 |
| Mean(SD) | Visit_5 | 24.9 ( 8.08) | 28.7 (15.27) |
| Median | Visit_5 | 24.0 | 23.5 |
| Min, Max | Visit_5 | 12, 42 | 10, 94 |
| **SGPT U/L** | | | |
| N | Visit_1 | 49 | 49 |
| Mean(SD) | Visit_1 | 38.6 (20.86) | 37.2 (18.56) |
| Median | Visit_1 | 33.0 | 33.0 |
| Min, Max | Visit_1 | 14, 116 | 17, 136 |
| N | Visit_5 | 49 | 48 |
| Mean(SD) | Visit_5 | 38.2 (20.62) | 39.9 (21.10) |
| Median | Visit_5 | 31.0 | 32.5 |
| Min, Max | Visit_5 | 14, 116 | 16, 106 |

**Table: Routine urine parameters**

| **Parameter** | **Visit** | **Amla extract (N=49)** | **Placebo (N=49)** | **Total (N=98)** |
| --- | --- | --- | --- | --- |
| **Appearance, n[%]** |  |  |  |  |
| Clear | Visit_1 | 26(53.1) | 14(28.6) | 40(40.8) |
| Clear | Visit_5 | 22(44.9) | 20(40.8) | 42(42.9) |
| Hazy | Visit_1 | 0( 0.0) | 1( 2.0) | 1( 1.0) |
| Hazy | Visit_5 | 0( 0.0) | 0( 0.0) | 0( 0.0) |
| NOT DONE | Visit_1 | 0( 0.0) | 0( 0.0) | 0( 0.0) |
| NOT DONE | Visit_5 | 0( 0.0) | 1( 2.0) | 1( 1.0) |
| Slightly Hazy | Visit_1 | 23(46.9) | 32(65.3) | 55(56.1) |
| Slightly Hazy | Visit_5 | 26(53.1) | 27(55.1) | 53(54.1) |
| Turbid | Visit_1 | 0( 0.0) | 1( 2.0) | 1( 1.0) |
| Turbid | Visit_5 | 0( 0.0) | 1( 2.0) | 1( 1.0) |
| **pH** | | | | |
| N | Visit_1 | 49 | 48 | 97 |
| Mean (SD) | Visit_1 | 5.8( 0.49) | 5.9( 0.55) | 5.9( 0.52) |
| Median | Visit_1 | 6.0 | 6.0 | 6.0 |
| Min, Max | Visit_1 | 5.0, 7.0 | 5.0, 7.5 | 5.0, 7.5 |
| N | Visit_5 | 48 | 48 | 96 |
| Mean (SD) | Visit_5 | 6.0( 0.47) | 5.9( 0.34) | 6.0( 0.41) |
| Median | Visit_5 | 6.0 | 6.0 | 6.0 |
| Min, Max | Visit_5 | 5.0, 7.5 | 5.5, 7.0 | 5.0, 7.5 |
| **Specific Gravity** | | | | |
| N | Visit_1 | 49 | 48 | 97 |
| Mean (SD) | Visit_1 | 1.0( 0.01) | 1.0( 0.01) | 1.0( 0.01) |
| Median | Visit_1 | 1.0 | 1.0 | 1.0 |
| Min, Max | Visit_1 | 1.0, 1.0 | 1.0, 1.0 | 1.0, 1.0 |
| N | Visit_5 | 48 | 48 | 96 |
| Mean (SD) | Visit_5 | 21.9( 144.91) | 1.0( 0.05) | 11.5( 102.47) |
| Median | Visit_5 | 1.0 | 1.0 | 1.0 |
| Min, Max | Visit_5 | 1.0, 1.005 | 1.0, 1.2 | 1.0, 1.005 |
| **Glucose, n[%]** | | | | |
| Detected | Visit_1 | 1( 2.0) | 3( 6.1) | 4( 4.1) |
| Detected | Visit_5 | 1( 2.0) | 2( 4.1) | 3( 3.1) |
| ND | Visit_1 | 48(98.0) | 45(91.8) | 93(94.9) |
| ND | Visit_5 | 47(95.9) | 46(93.9) | 93(94.9) |
| NOT DONE | Visit_1 | 0( 0.0) | 0( 0.0) | 0( 0.0) |
| NOT DONE | Visit_5 | 0( 0.0) | 1( 2.0) | 1( 1.0) |
| **Protein-mg/dl, n[%]** | | | | |
| Detected | Visit_1 | 6(12.2) | 7(14.3) | 13(13.3) |
| Detected | Visit_5 | 0( 0.0) | 3( 6.1) | 3( 3.1) |
| ND | Visit_1 | 43(87.8) | 41(83.7) | 84(85.7) |
| ND | Visit_5 | 48(98.0) | 45(91.8) | 93(94.9) |
| NOT DONE | Visit_1 | 0( 0.0) | 0( 0.0) | 0( 0.0) |
| NOT DONE | Visit_5 | 0( 0.0) | 1( 2.0) | 1( 1.0) |
| **Ketones, n[%]** | | | | |
| Detected | Visit_1 | 1( 2.0) | 1( 2.0) | 2( 2.0) |
| Detected | Visit_5 | 0( 0.0) | 0( 0.0) | 0( 0.0) |
| ND | Visit_1 | 48(98.0) | 47(95.9) | 95(96.9) |
| ND | Visit_5 | 48(98.0) | 48(98.0) | 96(98.0) |
| NOT DONE | Visit_1 | 0( 0.0) | 0( 0.0) | 0( 0.0) |
| NOT DONE | Visit_5 | 0( 0.0) | 1( 2.0) | 1( 1.0) |
| Blood, n[%] |  |  |  |  |
| Detected | Visit_1 | 8(16.3) | 14(28.6) | 22(22.4) |
| Detected | Visit_5 | 7(14.3) | 12(24.5) | 19(19.4) |
| ND | Visit_1 | 41(83.7) | 34(69.4) | 75(76.5) |
| ND | Visit_5 | 41(83.7) | 36(73.5) | 77(78.6) |
| NOT DONE | Visit_1 | 0( 0.0) | 0( 0.0) | 0( 0.0) |
| NOT DONE | Visit_5 | 0( 0.0) | 1( 2.0) | 1( 1.0) |
| **Bilirubin, n[%]** | | | | |
| ND | Visit_1 | 49( 100) | 48(98.0) | 97(99.0) |
| ND | Visit_5 | 48(98.0) | 48(98.0) | 96(98.0) |
| NOT DONE | Visit_1 | 0( 0.0) | 0( 0.0) | 0( 0.0) |
| NOT DONE | Visit_5 | 0( 0.0) | 1( 2.0) | 1( 1.0) |
| **Urobilinogen-mg/dL,n[%]** | | | | |
| ND | Visit_1 | 0( 0.0) | 0( 0.0) | 0( 0.0) |
| ND | Visit_5 | 3( 6.1) | 2( 4.1) | 5( 5.1) |
| NOT DONE | Visit_1 | 0( 0.0) | 0( 0.0) | 0( 0.0) |
| NOT DONE | Visit_5 | 0( 0.0) | 1( 2.0) | 1( 1.0) |
| Normal | Visit_1 | 49( 100) | 48(98.0) | 97(99.0) |
| Normal | Visit_5 | 45(91.8) | 46(93.9) | 91(92.9) |
| **Nitrite, n[%]** | | | | |
| Detected | Visit_1 | 0( 0.0) | 2( 4.1) | 2( 2.0) |
| Detected | Visit_5 | 2( 4.1) | 3( 6.1) | 5( 5.1) |
| ND | Visit_1 | 49( 100) | 46(93.9) | 95(96.9) |
| ND | Visit_5 | 46(93.9) | 45(91.8) | 91(92.9) |
| NOT DONE | Visit_1 | 0( 0.0) | 0( 0.0) | 0( 0.0) |
| NOT DONE | Visit_5 | 0( 0.0) | 1( 2.0) | 1( 1.0) |
| **WBC-μl, n[%]** | | | | |
| 0-1 | Visit_1 | 29(59.2) | 28(57.1) | 57(58.2) |
| 0-1 | Visit_5 | 16(32.7) | 17(34.7) | 33(33.7) |
| 1-2 | Visit_1 | 9(18.4) | 11(22.4) | 20(20.4) |
| 1-2 | Visit_5 | 16(32.7) | 13(26.5) | 29(29.6) |
| 10-15 | Visit_1 | 2( 4.1) | 0( 0.0) | 2( 2.0) |
| 10-15 | Visit_5 | 0( 0.0) | 0( 0.0) | 0( 0.0) |
| 15-20 | Visit_1 | 0( 0.0) | 1( 2.0) | 1( 1.0) |
| 15-20 | Visit_5 | 1( 2.0) | 1( 2.0) | 2( 2.0) |
| 2-3 | Visit_1 | 3( 6.1) | 4( 8.2) | 7( 7.1) |
| 2-3 | Visit_5 | 9(18.4) | 9(18.4) | 18(18.4) |
| 20-30 | Visit_1 | 0( 0.0) | 0( 0.0) | 0( 0.0) |
| 20-30 | Visit_5 | 0( 0.0) | 1( 2.0) | 1( 1.0) |
| 3-5 | Visit_1 | 5(10.2) | 1( 2.0) | 6( 6.1) |
| 3-5 | Visit_5 | 5(10.2) | 2( 4.1) | 7( 7.1) |
| 30-40 | Visit_1 | 0( 0.0) | 0( 0.0) | 0( 0.0) |
| 30-40 | Visit_5 | 0( 0.0) | 1( 2.0) | 1( 1.0) |
| 40-50 | Visit_1 | 0( 0.0) | 1( 2.0) | 1( 1.0) |
| 40-50 | Visit_5 | 0( 0.0) | 1( 2.0) | 1( 1.0) |
| 5-7 | Visit_1 | 0( 0.0) | 2( 4.1) | 2( 2.0) |
| 5-7 | Visit_5 | 0( 0.0) | 3( 6.1) | 3( 3.1) |
| 8-10 | Visit_1 | 1( 2.0) | 0( 0.0) | 1( 1.0) |
| 8-10 | Visit_5 | 1( 2.0) | 0( 0.0) | 1( 1.0) |
| NOT DONE | Visit_1 | 0( 0.0) | 0( 0.0) | 0( 0.0) |
| NOT DONE | Visit_5 | 0( 0.0) | 1( 2.0) | 1( 1.0) |
| **Epithelial Cells/hpf, n[%]** | | | | |
| 0-1 | Visit_1 | 21(42.9) | 21(42.9) | 42(42.9) |
| 0-1 | Visit_5 | 20(40.8) | 20(40.8) | 40(40.8) |
| 0-2 | Visit_1 | 0( 0.0) | 0( 0.0) | 0( 0.0) |
| 0-2 | Visit_5 | 1( 2.0) | 0( 0.0) | 1( 1.0) |
| 1-2 | Visit_1 | 4( 8.2) | 6(12.2) | 10(10.2) |
| 1-2 | Visit_5 | 4( 8.2) | 4( 8.2) | 8( 8.2) |
| 10-15 | Visit_1 | 1( 2.0) | 0( 0.0) | 1( 1.0) |
| 10-15 | Visit_5 | 0( 0.0) | 1( 2.0) | 1( 1.0) |
| 15-20 | Visit_1 | 0( 0.0) | 1( 2.0) | 1( 1.0) |
| 15-20 | Visit_5 | 1( 2.0) | 0( 0.0) | 1( 1.0) |
| 2-3 | Visit_1 | 14(28.6) | 9(18.4) | 23(23.5) |
| 2-3 | Visit_5 | 12(24.5) | 11(22.4) | 23(23.5) |
| 2-5 | Visit_1 | 0( 0.0) | 0( 0.0) | 0( 0.0) |
| 2-5 | Visit_5 | 0( 0.0) | 1( 2.0) | 1( 1.0) |
| 3-4 | Visit_1 | 0( 0.0) | 0( 0.0) | 0( 0.0) |
| 3-4 | Visit_5 | 1( 2.0) | 0( 0.0) | 1( 1.0) |
| 3-5 | Visit_1 | 7(14.3) | 6(12.2) | 13(13.3) |
| 3-5 | Visit_5 | 4( 8.2) | 3( 6.1) | 7( 7.1) |
| 5-7 | Visit_1 | 2( 4.1) | 4( 8.2) | 6( 6.1) |
| 5-7 | Visit_5 | 3( 6.1) | 3( 6.1) | 6( 6.1) |
| 8-10 | Visit_1 | 0( 0.0) | 1( 2.0) | 1( 1.0) |
| 8-10 | Visit_5 | 2( 4.1) | 5(10.2) | 7( 7.1) |
| NOT DONE | Visit_1 | 0( 0.0) | 0( 0.0) | 0( 0.0) |
| NOT DONE | Visit_5 | 0( 0.0) | 1( 2.0) | 1( 1.0) |
| **RBC/hpf, n[%]** | | | | |
| 0-1 | Visit_1 | 2( 4.1) | 3( 6.1) | 5( 5.1) |
| 0-1 | Visit_5 | 1( 2.0) | 0( 0.0) | 1( 1.0) |
| 1-2 | Visit_1 | 1( 2.0) | 2( 4.1) | 3( 3.1) |
| 1-2 | Visit_5 | 0( 0.0) | 1( 2.0) | 1( 1.0) |
| 15-20 | Visit_1 | 0( 0.0) | 1( 2.0) | 1( 1.0) |
| 15-20 | Visit_5 | 0( 0.0) | 0( 0.0) | 0( 0.0) |
| 5-7 | Visit_1 | 0( 0.0) | 0( 0.0) | 0( 0.0) |
| 5-7 | Visit_5 | 1( 2.0) | 0( 0.0) | 1( 1.0) |
| 8-10 | Visit_1 | 0( 0.0) | 1( 2.0) | 1( 1.0) |
| 8-10 | Visit_5 | 0( 0.0) | 0( 0.0) | 0( 0.0) |
| Detected | Visit_1 | 6(12.2) | 5(10.2) | 11(11.2) |
| Detected | Visit_5 | 4( 8.2) | 11(22.4) | 15(15.3) |
| ND | Visit_1 | 40(81.6) | 36(73.5) | 76(77.6) |
| ND | Visit_5 | 42(85.7) | 36(73.5) | 78(79.6) |
| NOT DONE | Visit_1 | 0( 0.0) | 0( 0.0) | 0( 0.0) |
| NOT DONE | Visit_5 | 0( 0.0) | 1( 2.0) | 1( 1.0) |
| **Casts, n[%]** | | | | |
| Detected | Visit_1 | 4( 8.2) | 1( 2.0) | 5( 5.1) |
| Detected | Visit_5 | 0( 0.0) | 0( 0.0) | 0( 0.0) |
| ND | Visit_1 | 45(91.8) | 47(95.9) | 92(93.9) |
| ND | Visit_5 | 48(98.0) | 48(98.0) | 96(98.0) |
| NOT DONE | Visit_1 | 0( 0.0) | 0( 0.0) | 0( 0.0) |
| NOT DONE | Visit_5 | 0( 0.0) | 1( 2.0) | 1( 1.0) |
| **Crystals, n[%]** | | | | |
| Detected | Visit_1 | 5(10.2) | 8(16.3) | 13(13.3) |
| Detected | Visit_5 | 2( 4.1) | 6(12.2) | 8( 8.2) |
| ND | Visit_1 | 44(89.8) | 40(81.6) | 84(85.7) |
| ND | Visit_5 | 46(93.9) | 42(85.7) | 88(89.8) |
| NOT DONE | Visit_1 | 0( 0.0) | 0( 0.0) | 0( 0.0) |
| NOT DONE | Visit_5 | 0( 0.0) | 1( 2.0) | 1( 1.0) |
| **Bacteria, n[%]** | | | | |
| Detected | Visit_1 | 18(36.7) | 26(53.1) | 44(44.9) |
| Detected | Visit_5 | 28(57.1) | 29(59.2) | 57(58.2) |
| ND | Visit_1 | 31(63.3) | 22(44.9) | 53(54.1) |
| ND | Visit_5 | 20(40.8) | 19(38.8) | 39(39.8) |
| NOT DONE | Visit_1 | 0( 0.0) | 0( 0.0) | 0( 0.0) |
| NOT DONE | Visit_5 | 0( 0.0) | 1( 2.0) | 1( 1.0) |
